# Accelerated vegetation succession but no hydrological change in a boreal fen during 20 years of recent climate change

**DOI:** 10.1002/ece3.7592

**Published:** 2021-05-02

**Authors:** Tiina H. M. Kolari, Pasi Korpelainen, Timo Kumpula, Teemu Tahvanainen

**Affiliations:** ^1^ Department of Environmental and Biological Sciences University of Eastern Finland Joensuu Finland; ^2^ Department of Geographical and Historical Studies University of Eastern Finland Joensuu Finland

**Keywords:** aapa mire, fen vegetation, hummock formation, remapping, resampling, rich fen, specialist species, water‐table depth

## Abstract

Northern mires (fens and bogs) have significant climate feedbacks and contribute to biodiversity, providing habitats to specialized biota. Many studies have found drying and degradation of bogs in response to climate change, while northern fens have received less attention. Rich fens are particularly important to biodiversity, but subject to global climate change, fen ecosystems may change via direct response of vegetation or indirectly by hydrological changes. With repeated sampling over the past 20 years, we aim to reveal trends in hydrology and vegetation in a pristine boreal fen with gradient from rich to poor fen and bog vegetation. We resampled 203 semi‐permanent plots and compared water‐table depth (WTD), pH, concentrations of mineral elements, and dissolved organic carbon (DOC), plant species occurrences, community structure, and vegetation types between 1998 and 2018. In the study area, the annual mean temperature rose by 1.0°C and precipitation by 46 mm, in 20‐year periods prior to sampling occasions. We found that wet fen vegetation decreased, while bog and poor fen vegetation increased significantly. This reflected a trend of increasing abundance of common, generalist hummock species at the expense of fen specialist species. Changes were the most pronounced in high pH plots, where *Sphagnum* mosses had significantly increased in plot frequency, cover, and species richness. Changes of water chemistry were mainly insignificant in concentration levels and spatial patterns. Although indications toward drier conditions were found in vegetation, WTD had not consistently increased, instead, our results revealed complex dynamics of WTD as depending on vegetation changes. Overall, we found significant trend in vegetation, conforming to common succession pattern from rich to poor fen and bog vegetation. Our results suggest that responses intrinsic to vegetation, such as increased productivity or altered species interactions, may be more significant than indirect effects via local hydrology to the ecosystem response to climate warming.

## Graphical abstract

We studied global change effects on vegetation and hydrology in a pristine boreal mire in a decadal time‐span. We repeated over 200 measurements of plant species cover, water‐table depth, and several water chemistry variables, after a marked shift to warmer conditions. We did not find significant changes in water chemistry, while remarkable vegetation changes were observed, suggesting that responses intrinsic to vegetation were more significant than indirect effects via local hydrology to the ecosystem response to recent warming.

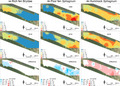

## 1 INTRODUCTION

Loss of biodiversity and ecosystem services due to human activities are a concern for many ecosystems globally. Mires (fens and bogs) are among the most severely endangered ecosystems, hosting a range of specialized biota. For example, in the European Red List of Habitats, 11 of 13 mire habitat types (85%) were assessed as threatened (Janssen et al., 2016). Mires are threatened by hydrological modifications, peat extraction, eutrophication, and climate change. Rich fens are of specific interest as they harbor high species richness and are important habitats for many rare and threatened species, for example, land snails and calcium‐tolerant brown mosses (Horsáková et al., 2018; Jiménez‐Alfaro et al., 2012). Rich fens require base‐rich groundwater supply that maintains high pH, and relatively poor availability of main nutrients (phosphorus and nitrogen). In polluted areas with high land‐use intensity, the still remaining rich fens have been rapidly losing their specialized plant species (Bergamini et al., 2009; Hájek et al., 2015; Kooijman, 2012) and underwent succession toward grasslands and shrublands (Hájek, et al., 2020). In boreal mires, water‐table may fall with projected climate change (Gong et al., 2012) and induce changes in vegetation and greenhouse gas fluxes (Kokkonen et al., 2019; Laine et al., 2019; Mäkiranta et al., 2018). Indeed, recent studies suggest widespread drying induced by climate change (Swindles et al., 2019; van Bellen et al., 2018), which is expected to cause reduction of carbon sink due to increased decomposition (Chaudhary et al., 2020; Hopple et al., 2020; Leifeld et al., 2019), while increased productivity may counteract this effect and increase the carbon sink capacity (Charman et al., 2013). Many studies have been limited to raised bogs along southern range of mires, and either to short‐term experiments of ecosystem functions or comprising millennial‐scale variation in paleoecological studies, while trends in mire vegetation during recent decades are poorly known, especially in fens.

The contemporary main distribution range of rich fens, like most mire types, spreads over sparsely populated regions in the boreal zone. While less severe history of utilization has left more sites in pristine conditions, the northern areas are not isolated from global change factors, specifically climate change, rise of atmospheric CO_2_, and deposition of nitrogen and pollutants. Therefore, it is alarming that studies of recent trends are nearly lacking in boreal‐rich fens, especially since certain gross‐scale biogeographic factors make northern fens particularly vulnerable. In glaciated northern areas, like most of Fennoscandia, rich fens have comparable vegetation and pH as those in calcareous nonglaciated areas but differ in mineral concentrations (Hájek, et al., 2021; Peterka et al., 2017). In Central‐European rich fens, for example, Ca concentrations generally range from ca. 10 mg/L to 50 mg/L, and up to 300 mg/L (Hájek et al., 2002). Moreover, both pH and calcium are reported to explain floristic variation along the poor to rich fen gradient (e.g., Hájek et al., 2002; Kutnar & Martinčič, 2003; Peterka et al., 2014). In Fennoscandia, however, calcareous conditions are rare, and correlation of calcium and vegetation is loose (Tahvanainen, 2004). Instead, all main base cations (Ca, K, Mg, and Na) tend to be equally important, and alkalinity is typically just high enough to buffer high pH. Therefore, Fennoscandian rich fens have weak buffering capacity, and they can be sensitive to changes in hydrology, deposition, and organic acid production in the carbon cycle that may shift pH and induce changes in vegetation.

Many mire types depend on specific climatic conditions, as perhaps best known from Finland (Luoto et al., 2004; Ruuhijärvi, 1960), Sweden (Rydin et al., 1999), Norway (Moen, 1985; Moen & Lillethun, 1999), and North‐West Russia (Kuznetsov, 2003), where fens prevail in the northern and alpine zones, and bogs have southern distribution. This pattern is affected by current climate change. In Finland, warming has been rapid after the late 1960s, since when the recent rate of increase of mean annual temperature has been ca. 0.3°C per decade (Mikkonen et al., 2015). While it is uncertain how vegetation responds to climate change, the climate‐hydrological correlation of contemporary pattern suggests that northern fens may start to develop into raised bogs. Indeed, paleo‐records from boreal mires have shown recent increase of *Sphagnum* mosses and shift toward ombrotrophic conditions during the 20th century, coinciding with warming and lengthening of the growing season (Loisel & Yu, 2013; Primeau & Garneau, 2021; Robitaille et al., 2021; van Bellen et al., 2018). Change from rich fen to *Sphagnum*‐dominated poor fen and bog vegetation means ecosystem‐scale shift and potential increase of carbon accumulation (Loisel & Bunsen, 2020; Loisel & Yu, 2013), and such processes can be triggered within few decades (Tahvanainen, 2011). On the other hand, in *Sphagnum*‐dominated poor fens, warming may induce shift to graminoid‐dominated vegetation (Dieleman et al., 2015).

Repeating historical studies provides a valuable tool for studying recent ecosystem changes (Hédl et al., 2017; Kapfer et al., 2017). Some resampling studies have documented changes in Fennoscandian mires over 5–60 years, but they have mainly focused on ombrotrophic bogs or poor fens in southern Sweden and Norway (Backéus, 1972; Gunnarsson & Flodin, 2007; Gunnarsson et al., 2002; Kapfer et al., 2011; Nordbakken, 2001). Recent changes in undrained bogs are mainly associated with nitrogen deposition, increased temperature, and drought (Gunnarsson & Flodin, 2007; Kapfer et al., 2011; Nordbakken, 2001). In SE Sweden, Pedrotti et al. (2014) found minor site‐level changes in bog vegetation, while their rich fen site had striking changes. Rich fen brown mosses had almost completely disappeared, *Sphagnum* hummocks had expanded, and pH declined. Similar changes were observed by Gunnarsson et al. (2000) in a rich fen site in Central Sweden. These studies demonstrate that significant changes from rich fen to poor fen and bog vegetation can, indeed, take place within just few decades, but question remains if such changes are expected in the northern latitudes of the main distribution range of rich fens. Furthermore, studies are missing the question if changes in hydrology and water chemistry are principal causes to changes in rich fen vegetation, which could be expected from the universal hydrochemical correlation of mire vegetation from poor to rich fens.

We repeated an intensive survey of water‐table depth, water chemical measurements, and over 200 vegetation plots after 20 years in a diverse and pristine aapa mire in the middle‐boreal zone, eastern Finland. The study site lies in a characteristic glaciated region with soft groundwater (Ca <5 mg/L), yet with a wide gradient from rich fen to poor fen, and bog vegetation. We aim to reveal if plant community structure or abundance of plant species and functional groups changed during the past 20 years, a period with marked shift in climate conditions in the study region, and if changes of vegetation and hydrology were connected. We examine if changes were spatially predictable, indicating trend of changing zonation, or irregular, either indicating over‐arching trend or small‐scale dynamics of microforms. In principle, our exploration of changes could result in four types of outcome: (a) no changes in either hydrology or vegetation, indicating ecosystem stability in the face of global change, (b) greater changes in hydrology than in vegetation, signifying biological inertia and resilience, i.e., delayed biotic responses to changing environmental drivers, (c) greater changes in vegetation, indicating impacts on biotic responses directly by global drivers, rather than indirectly via changes in local environmental conditions, or (d) simultaneous changes in both hydrology and vegetation, expected in terms of strict determinism between local environment and vegetation change, as driven by global change.

## 2 MATERIAL AND METHODS

### 2.1 The Study area

**FIGURE 1:**
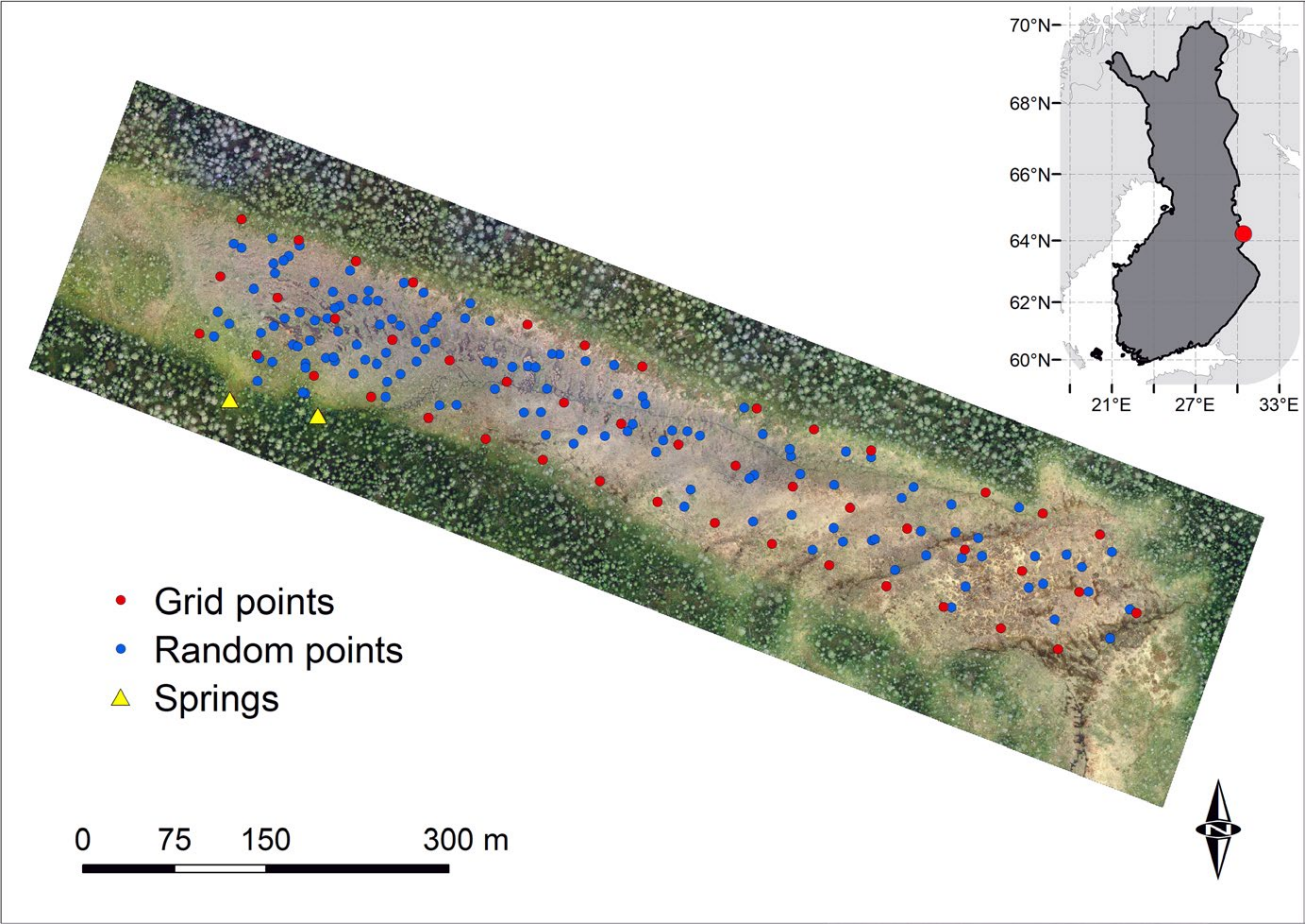
Location of the study site “Härkösuo” mire and sampling points. Random points refer to the vegetation plot locations that were randomly selected within grid squares in 1999. During the first sampling in 1998–1999, water samples were collected only from grid points, while vegetation and water‐table depth were measured in all points. In 2018, vegetation and water‐table depth were measured and water samples collected in each point. Near the springs, vegetation was assessed and water samples collected altogether in 15 additional plots with 5‐m interval during both samplings

The study area is located in the middle‐boreal zone, eastern Finland (N64°12′, E30°26′, 235 m a.s.l.), and within the Elimyssalo Nature Reserve (Figure 1). The area belongs to the southern aapa mire zone (Ruuhijärvi, 1960) and lies on Archean bedrock mainly formed of tonalite (Geological Survey of Finland, open data). The 20‐year annual average temperature was 0.9°C prior to first sampling (1978–1997) and 1.9°C prior to the second sampling (1998–2017). Annual precipitation sums were 612 and 658 mm for the same time periods, respectively (Finnish Meteorological Institute, updated ClimGrid dataset, see Aalto et al., 2016, Figure 2).

**FIGURE 2:**
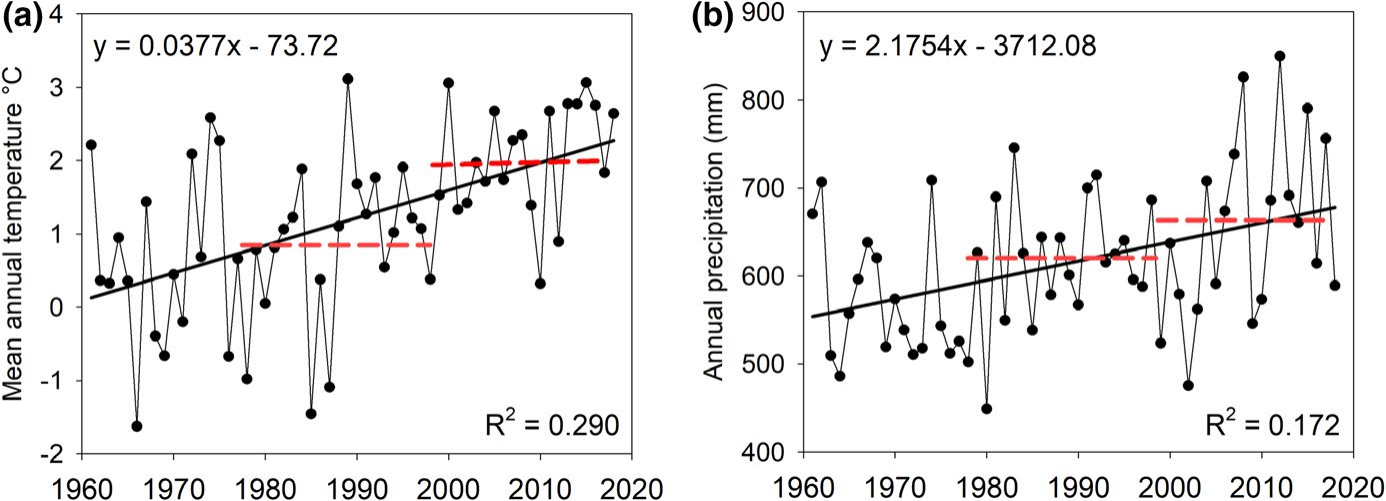
Climate patterns in the study area in 1961–2018, showing (a) mean annual temperature and (b) annual precipitation sum. Dashed lines indicate the 20‐year mean annual temperatures and precipitation sums before the first and the second sampling. Climate data were obtained from permutation‐based GlimGrid dataset created by Finnish Meteorological Institute (described in Aalto et al., 2016)

The study site “Härkösuo” mire is a narrow, sloping fen, approximately 1 km long and up to 150 m wide (0.171 km^2^). Location in a large Kuhmo‐Sotkamo drumlin field defines the east–west orientation of mire depression. In the western part of the mire, the input of minerogenic water from open springs and by diffuse percolation is relatively strong, while it diminishes toward east. The study area is in a pristine state with extremely limited human impact, not any drainage or forestry activities have taken place in the catchment area that could have affected the mire hydrology. The studied mire area has a wide gradient from rich to poor fen, and to marginal bog vegetation. Vegetation in rich fen areas mainly represents the alliances *Sphagno warnstorfii*‐*Tomentypnion nitentis* and *Stygio‐Caricion limosae* (Peterka et al., 2017), that is, the typical borealrich fen types. Poor fens have *Sphagno‐Caricion canescentis* and *Scheuchzerion palustris* communities. The “Härkösuo” mire was studied in detail for spatial variation of water chemistry and vegetation (Tahvanainen et al., 2002), and seasonal variation in 1998–1999 (Tahvanainen et al., 2003). Galanina and Heikkilä (2007) studied the zonation of vegetation types, using both Finnish and Russian classification system, and Kuznetsov et al. (2012) studied vegetation history and carbon accumulation.

### 2.2 Field sampling

A systematic grid of 71 water sampling points was established over the study area in 1998 with 50‐m point frequency (Tahvanainen et al., 2002) (Figure 1). Water samples were obtained also from two springs and from four additional points. The vegetation data of Tahvanainen et al. (2002) consists of 0.25 m^2^ plots at all water sampling points (*n* = 77), 148 plots randomly chosen within the grid squares, and 15 additional plots near the springs with 5‐m interval. In summer 2018, we repeated the water sampling and vegetation survey. Original vegetation plots had coordinates of 1‐m accuracy attached to the regular grid over the mire, and some wooden poles used to mark the grid points in 1998 remain in place. Using the coordinates of original grid points, we were able to obtain coordinates for the rest of the points. We relocated all plots using a Real‐Time Kinematic (RTK) system (TOPCON) and direct measurements with a tape measure. When needed, we checked from the old data whether the vegetation plot had been located in hummock or flark surface and adjusted the location accordingly. In several cases (*n* = 22), the relocation of original plots was verified by findings of wooden sticks in peat that were used to mark the plots in 1998–1999. In these 22 cases, the RTK‐located and true locations were separated on average by 2.0 m, which defines our relocation accuracy of the randomized plots inside the 50‐m grid cells.

In 2018, vegetation was resurveyed from 203 points in August to September 2018, focusing on the area with high continuous scatter of plots and covering the variation in vegetation (Figure 1). The cover (%) of each plant species was visually estimated at each 0.25 m^2^ plot. The observer of the 1999 data (T. Tahvanainen) took part in 2018 sampling, which aided adjusting subjective component of estimation and enhance comparability of the datasets. Water‐table depth (WTD), that is, the distance between water level and the top of bryophyte layer, was measured at each plot once in early July and once in mid‐August or mid‐September in 2018.

In 13–14 August 2018, we obtained 100‐ml water samples from all sampling points (*n* = 195). We used 50‐cm plastic pipe wells (diameter 32 mm) installed in late spring. The middle section (20 cm) of each well was perforated with 3.5‐mm holes. Each well was emptied at least twice during the summer, and once right before sampling, as the first water sample was often turbid or had dead insects. Some additional samples were collected in May, July, September 2018, and in August 2019 and 2020, to control seasonal and inter‐annual variation (Appendix S1).

### 2.3 Chemical analyses

Water samples were analyzed for pH, dissolved organic carbon (DOC), and 22 mineral elements. Most trace elements were under detection limits or near signals from deionized water controls and were omitted from analyses. Altogether, the repeated water chemistry data consists of pH, DOC and concentrations of Al, Ca, Fe, Mg, Mn, Na, and Si. Water pH was measured in the laboratory within 3 days from unaerated samples using a calibrated standard pH glass electrode (Consort). Prior to DOC and elemental analysis, water samples were filtered through 0.45 µm sterilized membrane filters (Pall Corporation). Samples for DOC analysis were stored at +5°C and analyzed with a multi N/C^®^ 2100 TOC analyzer (Analytik Jena AG) in 3 months. Samples for elemental analysis (10 ml) were treated with concentrated nitric acid (HNO_3_ TraceMetal™ grade, Fisher Chemicals) and stored frozen (−20°C) until analyzed in January or February 2019 by Inductively Coupled Plasma ‐ Mass Spectrometry (ICP‐MS) using a NeXION 350D ICP‐MS instrument (PerkinElmer Inc., Waltham, MA, USA). Multi‐element standard solution (TraceCERT^®^ Periodic table mix 1 for ICP, Sigma‐Aldrich) was used for the calibration of ICP‐MS.

### 2.4 Statistical analyses

#### 2.4.1 Water chemistry and water‐table depth (WTD)

For comparisons of water chemistry variables, the grid sampling points with data both from 1998 and 2018 were included (*n* = 48). We divided the water chemistry comparisons into four pH classes (>5.4, 4.6–5.4, 4.3–4.6, and <4.3) based on 1998 data, since different mire types may be differently susceptible to changes. Hereon, we refer to the classes as high pH (>5.4), intermediate (4.6–5.4), low (4.3–4.6), and extremely low pH (<4.3). Notice that the high pH ranges would reach pH 7.5 if samples had been aerated (see Tahvanainen & Tuomaala, 2003). We selected pH classes for subdivisions of the comparisons because variation along poor‐rich gradient in vegetation can be divided by pH values in an objective way (Bridgham et al., 1996; Tahvanainen, 2004), and since pH is a major chemical factor to potentially influence water chemistry; thus, pH classes may reveal changes over time. We tested the differences among the pH groups in pH, DOC, and mineral element concentrations between 1998 and 2018 with paired *t* tests. Since assumption of normality of paired differences did not hold in some cases, the results were confirmed with Wilcoxon signed rank test, and we report only results that showed significant *p*‐values with both tests. This applies also on comparisons of WTD and vegetation. All statistical analyses were performed in IBM SPSS Statistics (Version 25.0), if not mentioned otherwise.

The difference in mean WTD between years was tested with paired *t* test. We used two datasets: all repeated vegetation plots (*n* = 203) and plots with confirmed location (*n* = 22), to control for potential influence of relocation error. For the 2018 measurements, mean WTD of two‐point measurements (July and August/September) was calculated for each vegetation plot. In the old data, grid points had three measurements, while other plots had only one measurement of WTD. A fixed reference pole was in place at a *Scorpidium*‐flark in rich fen, in relation to which water‐table level (WTL) fluctuation was assessed in 1999 weekly monitoring (Tahvanainen et al., 2003), and we repeated WTL measurements on several occasions in 2018 to 2020 (Appendix S1).

Since individual measurements of WTD cannot account for potentially significant fluctuation, we also compared predicted and observed WTD values between years. First, we used external vegetation plot and WTD data (mainly from sites of Tahvanainen, 2004) to calculate a weighted average and a weighted standard deviation of WTD for each plant species (Appendix S2). Then, we calculated a predicted WTD value for each of our vegetation plots, based on species response values and species abundances (weighted average calibration; ter Braak & Barendregt, 1985). This procedure was done for both 1999 and 2018 data. Besides taking WTL fluctuation into account, we expect that comparisons of observed versus predicted WTD can indicate whether species composition was balanced with hydrological conditions. A good fit of observed and predicted WTD would indicate a balanced situation. If a mismatch were found, plant community could be offset from hydrological conditions, indicating imbalance and pressure toward a new steady state.

We used nonmetric multidimensional scaling (NMDS) to explore the plant community structure and correlations between plant community changes and changes of water chemistry and WTD. For each plot, we calculated the difference in ordination scores between 1998–1999 and 2018. This was done separately for first and second axis scores, which were first interpreted to represent independent directions of variation. Then, we calculated Pearson correlations between the differences of NMDS scores and differences of WTD and pH. Because pH was only measured from grid points in 1998, we only used those when calculating Pearson correlation for pH change. However, we included interpolated pH values (Tahvanainen et al., 2002) for unsampled locations in 1998 to visualize pH gradient in ordination biplot. NMDS was performed in PC‐ORD 7.04 (McCune & Mefford, 2016) using the Sorensen distance measure with default analysis options, orthogonal principal axes rotation, and randomization test with 249 random runs.

#### 2.4.2 Changes in the proportions of vegetation types

In order to aid and concretize interpretation of vegetation changes in terms of habitat type nomination, we applied an independently developed classification expert system EUNIS‐ESy (Chytrý et al., 2020). Expert systems are designed for assigning phytosociological relevés (or plots) to vegetation units of existing phytosociological classifications. The expert system of EUNIS habitat types assigns mire vegetation plots to habitat types based on species composition, corresponding to one or two alliances of the formalized classification of fen or bog vegetation (Chytrý et al., 2020; Peterka et al., 2017). The analysis was done in JUICE program Version 7 (Tichý, 2002). Applying this independent quantitative classification enables us to assess the significance of possible vegetation changes in a wider context. To test for significance of changes in proportion of vegetation types between the six categories, we used the chi‐square test (*df* 5) omitting the unclassified plots from the analysis.

#### 2.4.3 Species frequencies and cover of indicator species groups

We calculated the frequencies of occurrence of each species in 1999 and 2018 data and used Fisher's exact test to determine significance of change in plot frequency. We compared total covers of bryophyte species of three poor‐rich indicator groups (rich fen Bryidae, poor fen *Sphagnum*, and hummock *Sphagnum* species). The species forming each ecological group are the same as in Tahvanainen et al. (2002), except that *Sphagnum angustifolium*, *S. fallax*, and *S. flexuosum* were merged as *Sphagnum recurvum* agg., as we were uncertain of their correct identification in 1999. The differences of indicator species groups between years were tested with paired *t* tests. Nomenclature follows the Finnish Biodiversity Info Facility (https://laji.fi, 9.10.2020).

#### 2.4.4 Vegetation changes in pH classes

Within each pH class, Blocked Indicator Species Analysis (bISA; Dufrêne & Legendre, 1997) was employed to define characteristic species of 1999 and 2018 data. This analysis considers both frequency and abundance, and it is used to reveal which species had increased or decreased during the study period. We refer to significant indicator value for 1999 as decrease and to significant indicator value in 2018 as increase. The plot id was used as a block factor, and thus, testing focuses on pairwise differences of plots between years. Monte Carlo permutation tests with 4,999 runs were employed. These analyses were performed using PC‐ORD 7.04 (McCune & Mefford, 2016).

In addition, in each pH class we tested the differences in total cover of *Sphagnum* mosses, non‐sphagnaceous mosses, and vascular plants; species richness (S), Shannon diversity index (H), and the number of species of *Sphagnum*, non‐sphagnaceous mosses, and vascular plants between years using paired *t* tests.

#### 2.4.5 Spatial interpolation

Water chemistry variables most important to variation in vegetation (pH and sum of base cations: Ca, Mg, and Na), total covers of bryophyte indicator groups, and the differences between 1998–1999 and 2018 in these variables, were mapped by interpolation, to illustrate the potential spatial aspect of changes in water chemistry and vegetation gradients. For water chemistry variables, measured values from grid points were used for mapping the spatial patterns of 1999 and 2018 data, and for the 2018, data also interpolations with data from all points were performed to enable evaluation of representativeness of the grid point results (Appendix S1). For the WTD and bryophyte groups' data, measured values from all sampling points were included for both datasets.

We used ArcMap 10.6.1 program and ordinary kriging (OK) interpolation method to predict the values for unsampled locations. OK method and its application for spatial mapping have been discussed more in detail for example by Mueller et al. (2004) and Elumalai et al. (2017). In our study, weights for measured values were obtained from stable semivariogram models. With the spatial interpolations, we aimed at revealing if observed changes took place in certain mire areas or zones. If changes were spatially predictable, the result would indicate a trend of changing zonation, while if changes were spatially irregular, such result would rather indicate small‐scale dynamics. Certain results of WTD changes were potentially related to a Finnish forest reindeer (*Rangifer tarandus* ssp. *fennicus* Lönnb.) path that formed a shallow stream through the mire area, and we digitized the track for an overlay with WTD results.

## 3 RESULTS

### 3.1 Changes in water chemistry and water‐table depth (WTD)

In general, water chemistry had changed little over the study period, and the spatial patterns of pH and base cations remained grossly similar between 1998 and 2018 (Figure 5). In the sites where we had repeated sampling on several occasions in 1998–1999 and 2018–2020, variation was overlapping in pH, DOC, Al, Ca, Fe, Mg, Mn, Na, and Si (Appendix S1). In high pH plots, these variables did not differ significantly between 1998 and 2018, although average Ca concentration was notably higher and Fe concentration lower in 2018 (Table 1).

**TABLE 1:** Mean values of water chemistry and vegetation variables within pH classes in 1998–1999 and 2018 and paired *t* test results

	High pH		*p*	Intermediate pH		*p*	Low pH		*p*	Extr. low pH		*p*
	1998	2018		1998	2018		1998	2018		1998	2018	
pH	5.80	5.75		4.82	4.90		4.39	4.57		4.17	4.40	***
DOC (mg/L)	20.1	18.7		42.2	33.7		43.3	36.4	*	50.5	41.5	*
Al (mgl/L)	0.083	0.040		0.093	0.084		0.173	0.136		0.252	0.175	**
Ca (mg/L)	2.98	3.57		1.65	1.92		1.31	1.59		1.29	1.63	*
Fe (mg/L)	0.311	0.078		0.430	0.468		0.677	0.415	*	0.818	0.464	**
Mg (mg/L)	1.51	1.53		0.72	0.70		0.52	0.41		0.43	0.35	*
Mn (mg/L)	0.009	0.005		0.006	0.009		0.009	0.011		0.015	0.009	**
Na (mg/L)	2.70	2.45		1.50	1.23		1.37	0.97	**	1.15	0.96	*
Si (mg/L)	4.70	3.44		2.81	2.56		4.01	2.97	*	3.11	3.14	
*n*		10			9			11			18	
*Sphagnum* moss cover %	32	51	**	67	64		77	88		90	96	
Non‐sphagnaceous moss cover %	53	31	***	16	16		7	1	*	5	2	
Vascular plant cover %	26	35	**	25	29		19	18		21	21	
Species richness (S)	11.6	15.8	***	11.8	13.2	**	9.6	11.7	***	10.1	11.5	***
Shannon diversity index (H)	1.34	1.58	**	1.31	1.29		1.33	1.35		1.25	1.33	
Number of *Sphagnum* species	1.2	1.6	**	2.5	2.5		3.2	4.0	*	3.6	3.8	
Number of non‐sphagnaceous moss species	2.9	3.1		1.3	1.3		0.5	0.5		0.4	0.4	
Number of vascular plant species	7.2	10.2	***	7.6	8.8	**	5.4	6.6	**	5.9	6.9	***
*n*		53			52			45			53	

* *p* < .05.

** *p* < .01.

*** *p* < .001.

In extremely low pH plots, mean pH increased from 4.17 to 4.40 (paired *t* test: *p* < .001). Concentrations of DOC, Al, Fe, Mg, Mn, and Na were significantly lower in 2018, while Ca concentration was significantly higher. In low pH plots, concentrations of DOC, Fe, Na, and Si were significantly lower in 2018.

**FIGURE 3:**
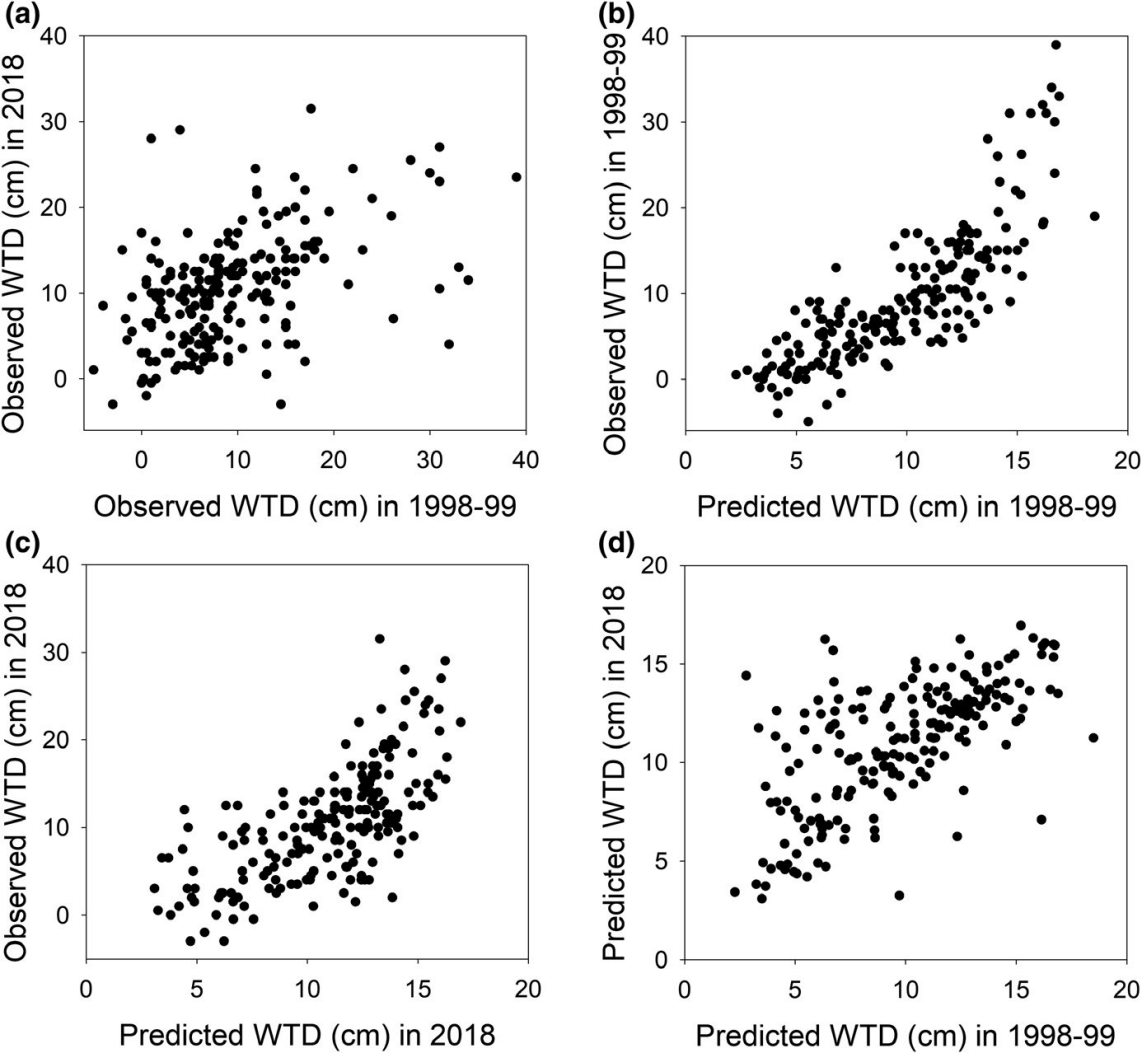
Comparisons of (a) mean water‐table depth (WTD) in 1998–1999 and 2018, (b) predicted and observed WTD in 1998–1999, (c) predicted and observed WTD in 2018, and (d) predicted WTD in 1998–1999 and 2018

Mean WTD values from 1998 to 1999 and 2018 correlated significantly but weakly (*r* = 0.461, *p* < .001), while no significant difference (*t* = −1.764, *p* = .079) was found (Figure 3). In the plots with confirmed locations (*n* = 22), this correlation was stronger (*r* = 0.703, *p* < .001), and no significant difference was found (*t* = −1.395, *p* = .178). The predicted and realized WTD values for 1998–1999 data had a strong significant positive correlation (*r* = 0.801, *p* < .001), while in 2018 this correlation was weaker, though still significant (*r* = 0.684, *p* < .001). Predicted values for 1998–1999 and 2018 correlated significantly but weakly (*r* = 0.661, *p* < .001). Average WTD was greater in 2018, but WTD values were not consistently greater in 2018 compared to 1998–1999. Interpolations showed spatial variation in WTD change (Figure 5). Increased WTD was found in the poor fen and mire margins, while some parts of rich fen and the most eastern part of the mire has become wetter. The observed Finnish forest reindeer track coincided with surface flow path (Appendix S1); it ran through a poor fen area with increased WTD and led to an area downstream where WTD had increased (Figure 5).

**FIGURE 4:**
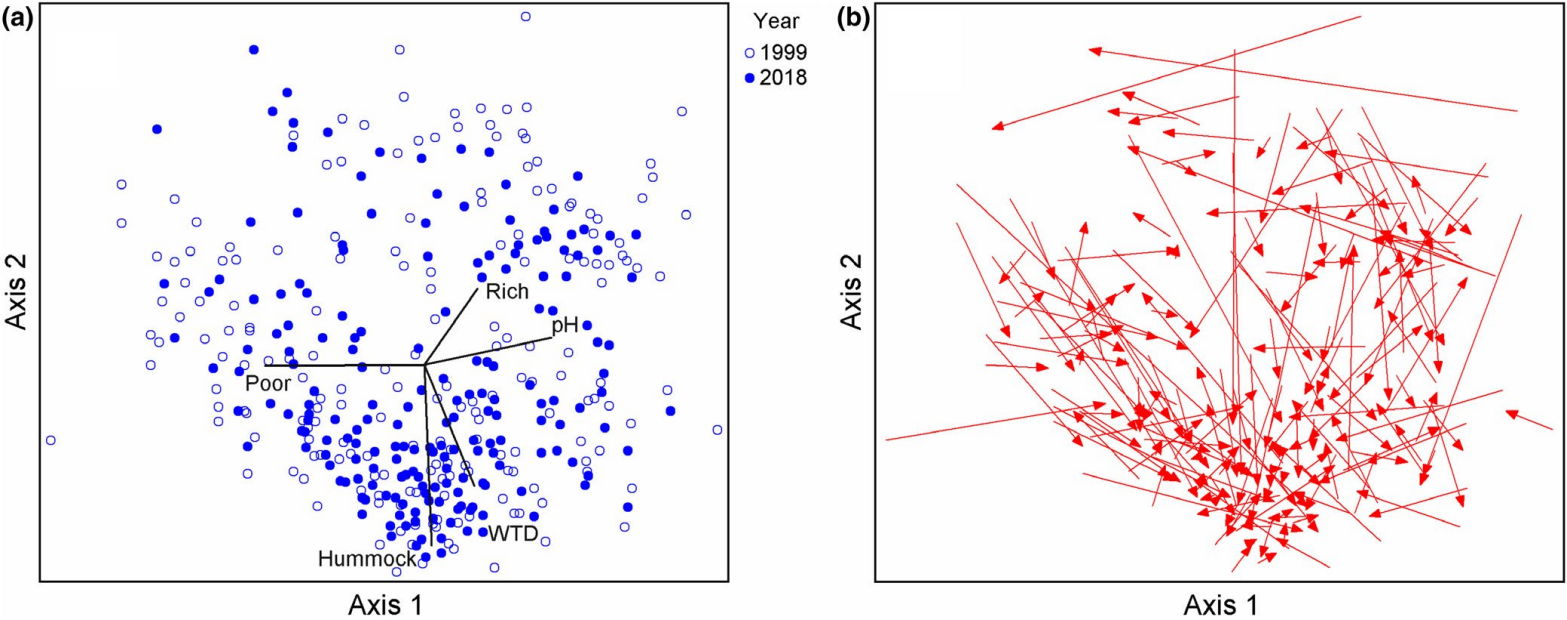
NMDS ordination of combined 1999 and 2018 vegetation plot data. (a) The first axis represents pH gradient, and the second axis water‐table depth (WTD) gradient. Poor, rich, and hummock refers to total covers of rich fen Bryidae, poor fen *Sphagnum*, and hummock *Sphagnum* species. (b) Plot locations have moved mainly in the same direction as the second axis representing WTD gradient

NMDS of combined plant community data (1999 and 2018) resulted a 2‐dimensional ordination (final stress value 22.73, Monte Carlo test, *p* = .004, 249 runs) and showed a clear orthogonal pattern of correlations with WTD and pH (Figure 4). The first axis represented the vegetation gradient from rich fen with high pH to acidic poor fen, and the second axis represented topographic variation from wet hollows to high hummocks. In the ordination space, plot locations moved mainly in the direction of the second axis, that is, along the WTD gradient. Change in NMDS plot score for both the first and second correlated significantly with WTD change (Axis 1: *r* = 0.287, *p* < .001; Axis 2: *r* = −0.525, *p* < .001), while no significant correlations were found for changes in NMDS plot scores and pH change.

### 3.2 Changes in the proportions of vegetation types

The expert system of EUNIS habitat types classified 88.9% of vegetation plots (Table 2). Significant difference in the proportions of habitat types was found between years (Chi^2^ = 14.631, *df*5, *p* = .012). The number of plots classified as calcareous quaking mire was markedly lower in 2018 compared to 1999, the proportion declining from 6.9% to 3% of all plots (57% decrease). The number of plots classified as noncalcareous quaking mire also declined, from 28.1% to 18.2%. Conversely, the number of plots classified as poor fen or bog increased remarkably. Poor fen plots increased from 3.4% to 8.9%, and the number of plots designated as bog vegetation increased from 30.5% to 38.9%. The number of plots classified as extremely rich moss‐sedge fen slightly increased from 16.3% to 18.7%, while intermediate fen and soft water spring mire occurrence did not differ between the surveys.

**TABLE 2:** Number of plots assigned to EUNIS habitat types in 1999 and 2018 vegetation plot data

EUNIS code	Habitat type	1998		2018		Change	
		*n*	%	*n*	%	Net	%
D4.1c	Calcareous quaking mire	14	6.9	6	3.0	−8	−57
D2.3a	Noncalcareous quaking mire	57	28.1	37	18.2	−20	−35
D4.1a	Extremely rich moss‐sedge fen	33	16.3	38	18.7	5	15
D2.2c	Intermediate fen and soft water spring mire	5	2.5	5	2.5	0	0
D2.2a	Poor fen	7	3.4	18	8.9	11	157
D1.1	Raised bog	62	30.5	79	38.9	17	27
	unclassified	25	12.3	20	9.9	−5	−19

Note

Vegetation plots were assigned to mire habitat types with the EUNIS‐Esy classification expert system (Chytrý et al., 2020). Mire habitat types correspond to one or two alliance of formalized classification of fen vegetation (Peterka et al., 2017), except D1.1 Raised bog, which correspond to alliances *Oxycocco microcarpi‐Empetrion hermaphroditi* (Nordhagen ex Du Rietz 1954 nom. conserv. propos.) and *Sphagnion medii* Kästner et Flössner 1933.

### 3.3 Total cover of bryophyte indicator groups and species frequencies

**FIGURE 5:**
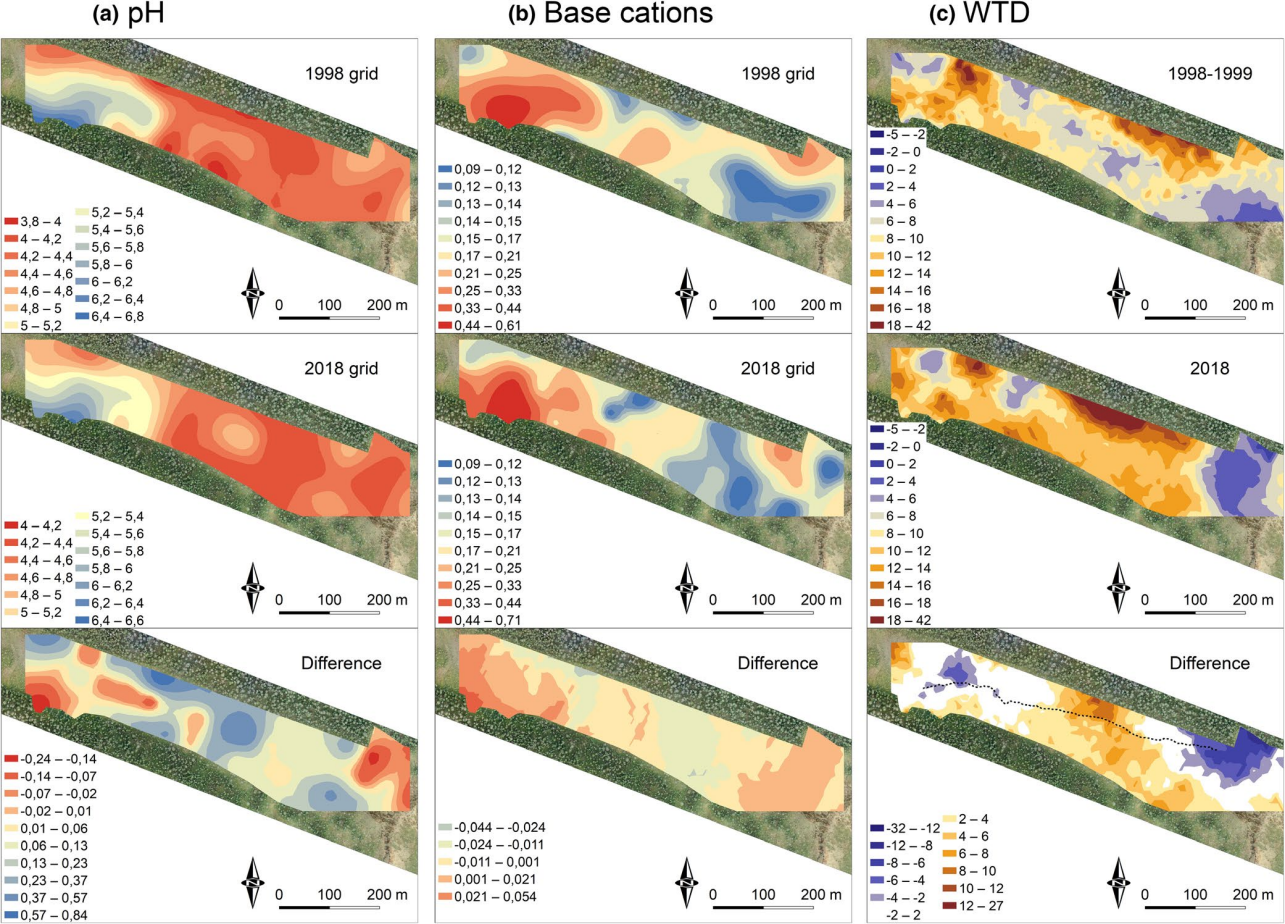
Spatial patterns of (a) pH, (b) base cations in meq/l (Ca, Mg, and Na), and (c) water‐table depth (WTD) in 1998–1999, 2018, and the difference between years. For pH and base cations, measured values from grid points (*n* = 48) were used to predict the values for unsampled points. For water‐table depth, measured values from all sampling points (*n* = 203) were included. The path created by Finnish forest reindeer is highlighted in the map showing the WTD difference. Ordinary kriging interpolation method with stable semivariogram model was used for all interpolations

**FIGURE 6:**
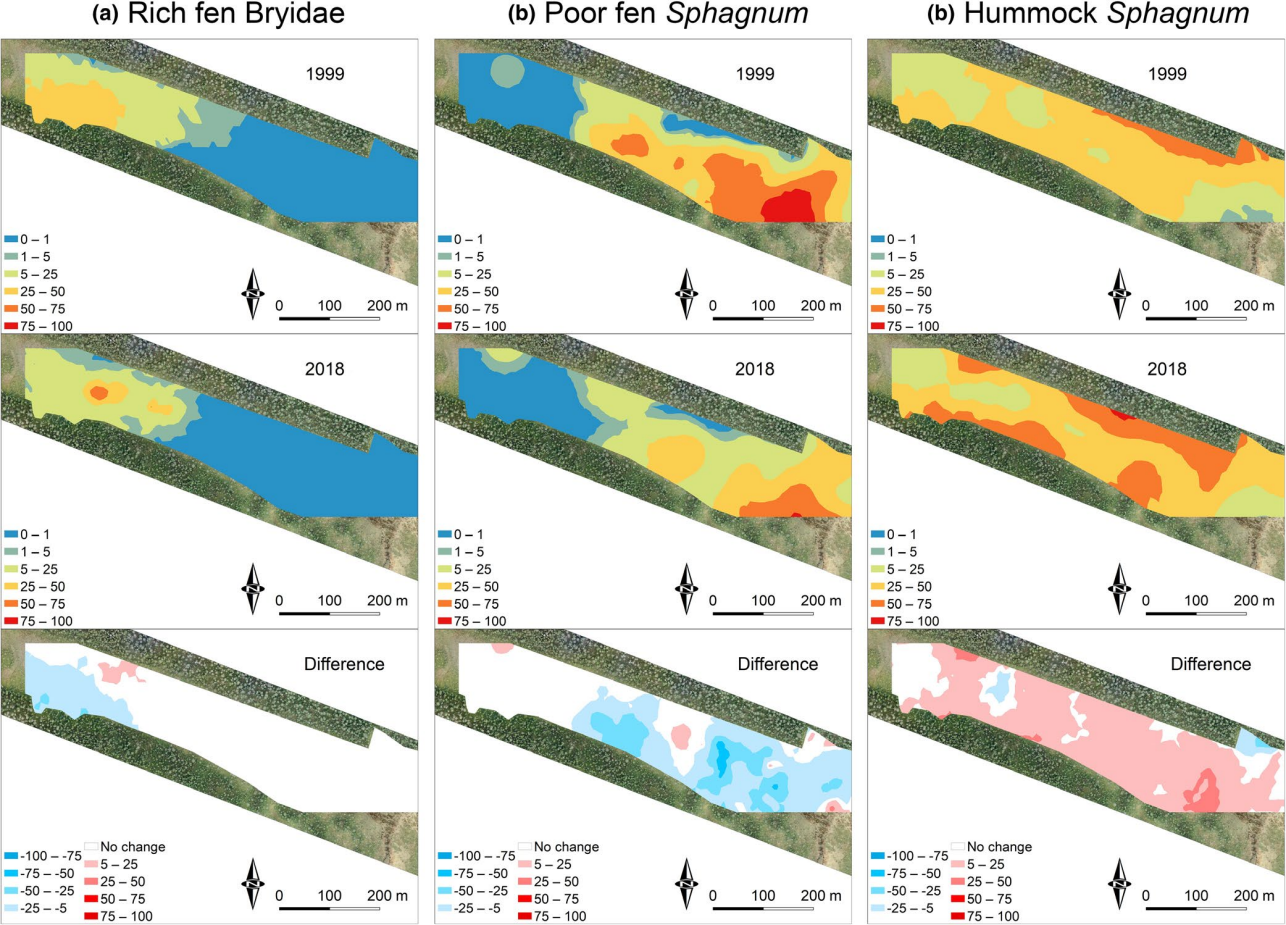
Spatial patterns of total covers of ecological groups of bryophyte species in 1999, 2018, and the difference between years: (a) rich fen Bryidae, (b) poor fen *Sphagnum*, and (c) hummock *Sphagnum* species. Spatial patterns were obtained from ordinary kriging interpolations, in which all vegetation plots (*n* = 203) were included

Total covers of bryophyte indicator groups showed significant differences between 1999 and 2018. On average, cover of rich fen Bryidae decreased from 14.7% to 9.6% (*t* = 3.192, *df* = 202, *p* = .002). Poor fen *Sphagnum* species decreased from 19.4% to 11.5% (*t* = 4.394, *df* = 202, *p* < .001), while the total cover of hummock *Sphagnum* species increased from 27.3% to 37.5% (*t* = −4.300, *df* = 202, *p* < .001). Although significant changes were detected, the distribution patterns of rich fen Bryidae and poor fen *Sphagnum* mosses followed closely that of pH and main cations in both datasets (Figures 5 and 6). Interpolation of spatial patterns of hummock *Sphagnum* species showed that most changes took place in the marginal mire areas.

In total, 128 species were found on the plots, 110 in 1999 and 112 in 2018. While the total number of species stayed nearly constant, 16 species were only met in 1999 and 18 only in 2018. Altogether, 26 species showed significant change (*p* < .05) in plot frequency (Table 3). Among these, 21 species increased significantly, mainly including common generalist species (*Andromeda polifolia, Drosera rotundifolia, Eriophorum vaginatum,* *Vaccinium oxycoccos*). Among increased mosses, hummock species (*Sphagnum recurvum* agg.*, S. fuscum, S. medium* coll.*, S*. *warnstorfii*) had experienced the largest increase in plot frequency. Fen specialists *Trichophorum alpinum, Sphagnum annulatum,* and *S*. *subfulvum* were among significantly decreased species.

**TABLE 3:** A list of species occurring in plots and their changes in plot frequency between 1999 and 2018

Species	Frequencies		Change			bISA			
	1998	2018	Net	%	*p*	High	Interm.	Low	Extr. low
Bryophytes									
*Aneura pinguis*	3	7	4	133		–	–	–	–
*Aulacomnium palustre*	40	36	−4	−10		–	–	–	–
*Bryum weigelii*	6	1	−5	−83		↓	–	–	–
*Campylium stellatum*	16	14	−2	−13		–	–	–	–
*Cinclidium stygium*	3	3	0	0		–	–	–	–
*Dicranum bonjeanii*	11	21	10	91	*	↑	–	–	–
*D. undulatum*	3	6	3	100		–	–	–	–
*Drepanocladus trifarius*	3	0	−3	−100		–	–	–	–
*Hylocomiastrum umbratum*	5	1	−4	−80		–	–	–	–
*Hylocomium splendens*	1	3	2	200		–	–	–	–
*Loeskypnum badium*	41	39	−2	−5		–	–	–	–
*Mylia anomala*	11	14	3	27		–	–	–	–
*Paludella squarrosa*	12	9	−3	−25		–	–	–	–
*Philonotis fontana*	6	5	−1	−17		–	–	–	–
*Plagiomnium ellipticum*	3	2	−1	−33		–	–	–	–
*Pleurozium schreberi*	18	20	2	11		–	–	–	–
*Pohlia* cf. *nutans*	2	8	6	300		–	–	–	–
*P. strictum*	8	15	7	88		–	–	–	–
*Rhizomnium magnifolium*	5	1	−4	−80		–	–	–	–
*R. pseudopunctatum*	6	4	−2	−33		–	–	–	–
*Sarmentypnum sarmentosum*	8	4	−4	−50		–	–	–	–
*Scapania paludosa*	5	7	2	40		–	–	–	–
*Scorpidium revolvens*	5	3	−2	−40		–	–	–	–
*S. scorpioides*	11	7	−4	−36		–	–	–	–
*Sphagnum annulatum*	21	9	−12	−57	**	–	–	↓	–
*S. balticum*	33	34	1	3		–	–	–	–
*S. contortum*	0	3	3			–	–	–	–
*S. fuscum*	60	77	17	28	*	–	–	↑	–
*S. jensenii*	44	46	2	5		–	–	–	–
*S. lindbergii*	4	3	−1	−25		–	–	–	–
*S. medium* coll.	64	95	31	48	***	–	–	↑	↑
*S. majus*	26	20	−6	−23		–	–	–	–
*S. papillosum*	51	50	−1	−2		–	–	–	–
*S. platyphyllum*	13	8	−5	−36		–	↓	–	–
*S. recurvum* agg.	109	145	36	33	***	–	↑	↑	↑
*S. rubellum*	4	2	−2	−50		–	–	–	–
*S. russowii*	29	26	−3	−10		–	–	–	↓
*S. subfulvum*	10	3	−7	−70	*	–	–	–	–
*S. subsecundum*	7	5	−2	−29		–	–	–	–
*S. tenellum*	5	0	−5	−100		–	–	–	–
*S. warnstorfii*	49	64	15	31	*	↑	–	–	–
*Straminergon stramineum*	26	43	17	65	*	–	↑	–	–
*Tomentypnum nitens*	5	13	8	160	*	–	–	–	–
*Warnstorfia fluitans*	10	10	0	0		–	–	–	–
**Vascular plants**									
*Andromeda polifolia*	136	165	29	21	***	–	↑	↑	–
*Angelica sylvestris*	1	10	9	900	*	↑	–	–	–
*Avenella flexuosa*	0	3	3			–	–	–	–
*Betula nana*	83	85	2	2		–	–	–	–
*Calluna vulgaris*	24	31	7	29		–	–	–	–
*Carex chordorrhiza*	0	5	5			–	–	–	–
*C. dioica*	9	7	−2	−22		–	–	–	–
*C. globularis*	5	5	0	0		–	–	–	–
*C. lasiocarpa*	102	106	4	4		–	–	–	–
*C. limosa*	57	40	−17	−30	*	–	–	–	↓
*C. pauciflora*	44	72	28	64	**	–	–	↑	↑
*C. rostrata*	79	82	3	4		–	–	–	–
*Chamaedaphne calyculata*	18	21	3	17		–	–	–	–
*Dactylorhiza maculata*	3	9	6	200		–	–	–	–
*D. majalis* ssp*. lapponica*	5	2	−3	−60		–	–	–	–
*Drosera anglica*	17	9	−8	−47		–	↓	–	–
*D. rotundifolia*	54	122	68	126	***	↑	↑	–	↑
*Empetrum nigrum*	59	74	15	25	*	↑	–	–	↑
*Epilobium* cf. *palustre*	2	4	2	100		–	–	–	–
*Equisetum fluviatile*	36	36	0	0		–	–	–	–
*Eriophorum latifolium*	10	10	0	0		–	–	–	–
*E. vaginatum*	69	121	52	75	***	↑	–	↑	↑
*Filipendula ulmaria*	3	4	1	33			–	–	–
*Lysimachia europaea*	18	31	13	72	*	–	–	–	–
*Melampyrum pratense*	9	0	−9	−100	**	–	–	–	–
*Menyanthes trifoliata*	31	44	13	42	*	↑	–	↓	–
*Molinia caerulea*	30	47	17	57	**	↑	–	–	–
*Moneses uniflora*	0	3	3			–	–	–	–
*Pedicularis sceptrum‐carolinum*	5	4	−1	−20		–	–	–	–
*Rhododendron tomentosum*	4	6	2	50		–	–	–	–
*Rhynchospora alba*	10	22	12	120	*	–	↑	–	–
*Rubus chamaemorus*	27	30	3	11		–	–	–	–
*S. myrtilloides*	4	0	−4	−100		–	–	–	–
*S. phylicifolia*	0	5	5			–	–	–	–
*Scheuchzeria palustris*	64	98	34	53	***	↑	↑	–	–
*Selaginella selaginoides*	12	27	15	125	**	↑	–	–	–
*Solidago virgaurea*	3	3	0	0		–	–	–	–
*Tofieldia pusilla*	21	28	7	33		–	–	–	–
*Trichophorum alpinum*	53	16	−37	−70	***	↓	↓	↓	–
*T. cespitosum*	46	70	24	52	**	–	↑	–	–
*Vaccinium microcarpum*	11	4	−7	−64		–	–	–	–
*V. oxycoccos*	124	160	36	29	***	–	–	–	–
*V. uliginosum*	20	19	−1	−5		–	–	–	–
*V. vitis‐idaea*	6	3	−3	−50		–	–	–	–

Note

Species with at least three occurrences in 1999 and/or 2018 are included. Fisher's exact test significance is indicated for tests of species frequencies (*p < .05, **p < .01, ***p < .001). Blocked Indicator Species Analysis (bISA) was used to define species association within each pH class to 1999 and 2018 datasets, and species with significant (*p* < .05) indicator values are marked with arrows, as according to change direction from 1999 to 2018.

### 3.4 Vegetation changes in pH classes

In high pH plots, species richness and Shannon diversity index (H) were significantly higher in 2018 (Table 1). *Sphagnum* mosses and vascular plants were significantly more abundant in 2018, both in total cover and in species number, while the cover of non‐sphagnaceous mosses was significantly lower. *Trichophorum alpinum* and *Bryum weigelii* decreased significantly (bISA) (Table 3). Several species had increased from 1999, including species characteristic of rich fen hummocks (*Dicranum bonjeanii,* and *Sphagnum warnstorfii*), and generalist bog species (*Drosera rotundifolia, Trichophorum cespitosum, Empetrum nigrum,* and *Eriophorum vaginatum*).

In intermediate pH plots, overall species richness and the number of vascular plant species were significantly higher in 2018. Fen specialist species *Sphagnum platyphyllum,* and *Trichophorum alpinum* decreased significantly, as did *Drosera anglica*, a species confined to wet microsites. Instead, mainly generalist bog species (*Sphagnum recurvum* agg., *Andromeda polifolia, Drosera rotundifolia*, *Scheuchzeria palustris*, and *Trichophorum cespitosum*) increased (bISA).

In low pH plots, species richness of all plants and of vascular plants and *Sphagnum* species were significantly higher in 2018. Total cover of non‐sphagnaceous mosses was significantly lower in 2018. Fen specialist species *Sphagnum annulatum, Menyanthes trifoliata,* and *Trichophorum alpinum* decreased significantly, while many generalist bog species (*Sphagnum fuscum, S*. *medium* coll.*, S*. *recurvum* agg.*, Andomeda polifolia, Carex pauciflora, Eriophorum vaginatum*) significantly increased (bISA). In extremely low pH plots, species richness and number of vascular plant species were significantly higher in 2018. *Carex limosa* and *Sphagnum russowii* decreased, while many generalist bog species (*Sphagnum medium* coll., *S*. *recurvum* agg., *Drosera rotundifolia, Empetrum nigrum,* and *Eriophorum vaginatum*) increased (bISA).

## 4 DISCUSSION

### 4.1 Hydrochemical and micro‐topographical changes

In mires, hydrological conditions have tight connection to vegetation patterns and, hence, hydrological changes could drive vegetation changes by impact on water quality or water‐table depth (WTD). Most importantly, pH, alkalinity, and base cation concentrations depend on input of minerogenic water and correlate with the gradient from rich fen to poor fen and bog vegetation (Gorham & Janssens, 1992; Malmer, 1986; Tahvanainen, 2004). Second major gradient in mire vegetation follows the micro‐topography and is measured by WTD (Økland 2001; Økland, 1990; Malhotra et al., 2016). It reflects the interplay of vegetation and hydrology and is manifested by the occurrence of hummocks, lawns, carpets, and hollows. These two main gradients appeared in our study site and were expected to govern variation and temporal changes in plant community structure. In NMDS ordination, vegetation plot locations had moved mainly in the direction of WTD gradient, and changes in NMDS scores correlated significantly with WTD change, but not with pH change. After 20 years of first sampling, pH and concentrations of base cations (Ca, Mg, and Na) remained at the same levels, and the spatial patterning of water chemical variation remained nearly the same, indicating persistent input of minerogenic groundwater. Apart from differences in some trace elements, differences in water chemistry were generally small, and where evident, they could at least partly be contributed to effects of short‐term weather conditions. Comparisons of all observations over 20 years (1998–1999 and 2018–2020) from four monitoring sites showed overlapping inter‐annual variation in all water chemical variables, as well as in WTD (Appendix S1).

Opposite to our expectation of acidification and to findings from Swedish rich fens where lowering of pH was observed (Gunnarsson et al., 2000; Pedrotti et al., 2014), we found no significant changes in the rich fen. Instead, we found slightly higher pH in the poor fen. In addition, concentrations of some mineral elements (Al, Fe, Mg, Mn, and Na) were significantly lower in 2018 in the poor fen. Tahvanainen et al. (2003) found that water‐table level (WTL) was stable in the rich fen and more variable in the poor fen, and this difference was apparent from our observations as well (Appendix S1). More unstable hydrological conditions in the poor fen likely explain the observed differences in water chemistry. These may have resulted from multitude of short‐term effects, such as alterations of redox conditions and mineralization of peat (Mettrop et al., 2015), impacts on solubility and chemical partitioning as affected by changes in pH and DOC (Watmough & Orlovskaya, 2015), vertical water flow (Siegel et al., 1995), plant nutrient uptake (Mettrop et al., 2015), or plant microbial interactions (Jassey et al., 2013), during the warm conditions before 2018 sampling. The fall of Fe concentration possibly resulted from increased aeration and oxidation from soluble ferrous (Fe^2+^) to insoluble ferric (Fe^3+^) iron (Schwertmann, 1991; Wang et al. 2017). In contrast, Ca concentrations in poor fen sites were slightly higher in 2018. Similar rise of Ca concentration was found by Mettrop et al. (2015) in poor fen mesocosms in response to drought and inundation treatment. Increased DOC concentration could be expected if input of groundwater had declined (Sallantaus, 2006) or due to increased DOC production (Fenner et al., 2007), but this was not realized in our study area. Instead, the DOC concentrations decreased, which may have resulted from increased decay of DOC due to warmer soil conditions (Delarue et al., 2014) or association of DOC removal with iron oxidation (Wang et al., 2017).

Our observations of water chemistry differences are hardly conclusive, and the four monitoring sites showed overlapping ranges over the 20‐year period, indicating that the observed differences were not persistent. The similarity of water chemical variation in datasets 20 years apart indicates that no remarkable water chemical changes had taken place. However, it is possible that significant fluctuation of water chemistry took place during the 20‐year period between the sampling campaigns and affected vegetation development. Schweiger and Beierkuhnlein (2017) found that increase of *Sphagnum* mosses over spring fens took place in the 20th century, due to acid deposition and continued after geochemical recovery. They concluded that *Sphagnum*‐engineered vegetation succession proceeded independently of water pH once it was started by the period of acidification.

In contrast to the stability and spatial predictability of the hydrochemical gradient structure, the micro‐topographical variation appeared highly dynamic. In the monitoring site with a fixed reference pole, the whole range of water‐level variation was merely 4 cm, thus, proving that the *absolute* water‐table level had not changed in the rich fen during the 20‐year period. However, WTD showed only a weak correlation between the 1998–1999 and 2018 data. As mentioned, summer 2018 was warm and dry, but WTD values were not consistently greater compared to 1998–1999, and the four monitoring sites had overlapping ranges through the 20‐year period. Increased precipitation and sustained groundwater input from catchment were apparently enough to prevent water level from falling despite increased evaporation. Instead of overall drying trend, interpolation of WTD difference showed spatial variation over the study area (Figure 5). Poor fen and mire margins showed signs of drying, while rich fen and a poor fen area in eastern part of the mire became wetter. Drying in the mire margins corresponds to the increase of hummock *Sphagnum* species and likely reflects increasingly progressive hummock growth, rather than falling of WTL (Belyea & Clymo, 2001). In addition, we found clear paths with hoof marks of Finnish forest reindeer (*Rangifer tarandus ssp. fennicus* Lönnb.), which may have changed flow patterns and contributed to drive WTD changes in certain locations.

The predicted and observed WTD values for 1998–1999 data had a strong positive correlation, with few deviant values at high WTD. In other words, species occurred close to their expected conditions in relation to WTD. This correlation was markedly weaker in 2018 data, which may indicate that while vegetation had changed, the characteristic water‐table depth was not yet achieved. Correspondingly, there was no overall difference in WTD to indicate drying, while hummock vegetation had increased. Such imbalance between species composition and WTD could be explained by the increase of generalist species that have wide WTD niche. Hummock species have generally wider responses to WTD (Tahvanainen & Tolonen, 2004), and as hummock species had increased, the species indication of WTD became vaguer. A part of changes in WTD may have resulted from weather conditions and error in relocation of plots, as WTD varies considerably in small distances according to micro‐topography (Belyea & Clymo, 2001). Indeed, the correlation of WTD between the 1998–1999 and 2018 data was stronger for the sites with confirmed exact location than in the whole data. However, that would not explain the weakening correlation between expected and realized WTD, nor the overall shift toward hummock vegetation. In conclusion, we find that a mismatch has developed between WTD and vegetation that probably reflects imbalance and ongoing changes in plant communities.

### 4.2 Habitat‐generalist species takeover and decline of rich fen vegetation

Rich fens host unique species assemblages and are among the most threatened habitat types in Europe (Janssen et al., 2016). In Finland, conversion of mire habitats for forestry and agricultural land has been the main reason for degradation of rich fens (Kontula & Raunio, 2019). Although extensive forestry drainage campaigns have ceased after the 1990s, it is questionable if pristine hydrology protects rich fens, as the northern areas are experiencing pronounced climate warming (Jansen et al., 2020; Kivinen et al., 2017). While impacts of drainage are well known, our study offered a unique opportunity to explore changes in a pristine mire catchment. Indeed, we found trends of increasing abundance of common generalist species at the expense of rich fen specialists and of hummock species at the expense of wet‐microhabitat species. These trends were most clearly apparent from bryophyte indicator groups, as we found increase of hummock *Sphagnum* species, while rich fen Bryidae and poor fen *Sphagnum* species declined in their total cover. In addition, species that significantly increased in plot frequency throughout the study area (e.g., *Carex pauciflora, Drosera rotundifolia, Empetrum nigrum, Eriophorum vaginatum, Scheuchzeria palustris, Trichophorum cespitosum*, and *Vaccinium oxycoccos*) comprise some of the most common species in many types of mires in Fennoscandia, both fens and bogs. Observed increase of species richness also mainly reflected the increase of these generalist vascular plants and of hummock *Sphagnum* species in rich fen vegetation.

Several originally abundant‐rich fen species declined over 50% (*Warnstorfia sarmentosa*, *Sphagnum subfulvum*, *Trichophorum alpinum*), and many rich fen species that were already rare in the 1998 (*Bryum weigelii, Calliergon cordifolium, Cinclidium subrotundum, Drepanocladus trifarius, Hylocomiastrum umbratum, Rhizomnium magnifolium,* and *Salix myrtilloides*) disappeared or drastically declined (80% or more). Some rich fen species persisted with no significant decline (*Campylium stellatum, Loeskypnum badium, Paludella squarrosa*, and *Eriophorum latifolium*), and some rich fen species had significantly increased (*Dicranum bonjeanii*, *Sphagnum warnstorfii, Tomentypnum nitens, Angelica sylvestris, Selaginella selaginoides,* and *Tofieldia pusilla*). Remarkably, all the increased rich fen species occupy low hummocks and their increasing trend therefore complies to the general increase of hummock vegetation.

The winners and losers of the past 20‐year period included plant species with different niches and overall interpretation of vegetation change is complicated. Application of an external, novel approach of classification with expert system (Chytrý et al., 2020) showed significant overall change of designation of plots to habitat types. Most frequently, plots shifted in classification to more dry and oligotrophic mire types (poor fen and bog) in 2018, while the wet habitat type designations (calcareous and noncalcareous quaking fens) decreased. Among the rich fen types, the wet type Quaking calcareous fen (alliance *Stygio‐Caricion limosae*) decreased by 57%, and it is characterized by *S. scorpioides, Cinclidium stygium*, and *Drepanocladus trifarius*, all of which had declined in our study area. These changes of vegetation types indicate a general shift toward drier vegetation type. Indeed, the number of plots classified as extremely rich moss‐sedge fen (alliance *Sphagno warnstorfii‐Tomentypnion nitensis*) slightly increased (15%), in line with the increased plot frequency of rich fen hummock species *S. warnstorfii* and *T. nitens*. All rich fen types are assessed as critically endangered (CR) in southern half of Finland, where our study site is located, and mainly threatened by anthropogenic disturbances (Kontula & Raunio, 2019). Our study shows that a pristine site is not safe from changes either and this warrants extra attention on state of rich fens as endangered habitats.

For the most part, our results correspond to findings of Gunnarsson et al. (2000) and Pedrotti et al. (2014) from Swedish rich fens. Both studies found decrease of rich fen brown mosses after 50 years. Furthermore, Pedrotti et al. (2014) found expansion of hummocks and increased abundance of shrubs, and the changes were consistent with reduced pH. They considered that drying had been more important to vegetation change, while they did not have repeated measurements of WTD. The loss of habitat‐specialist species due to anthropogenic change, while generalist species benefit, is currently a common phenomenon among many species groups and habitats (e.g., Ibarra & Martin, 2015; Polus et al., 2007). Recent decrease of habitat‐specialist bryophytes has been observed in springs in southern Finland (Heino et al., 2005; Juutinen, 2011). Although the species lists only partially overlap between our studies, the results were similar in revealing the increase of generalists at the expense of specialists among bryophytes. Loss of rich fen bryophytes has also been reported from base‐rich fens in Czech Republic (Hájek et al., 2015), Switzerland (Bergamini et al., 2009), and the Netherlands (Kooijman, 1992, 2012). In these cases, decline of specialist species has been attributed to nutrient enrichment, either phosphorus fertilization or atmospheric nitrogen deposition, and increase of competitive nutrient‐demanding species. In the Netherlands for example, the rich fen moss *Scorpidium scorpioides* has been taken over by *Calliergonella cuspidata* and *Sphagnum squarrosum* under increased nutrient availability (Kooijman, 2012). In addition, Hájek et al. (2015) found increased abundance of *Sphagnum recurvum* agg., which was observed throughout our study area as well. However, above‐mentioned studies were conducted in central Europe, with high nitrogen pollution (Bragazza et al., 2004; Harmens et al., 2015). While nitrogen deposition is suggested to influence mire vegetation also from southern‐boreal bogs to arctic tundra (Choudhary et al., 2016; Limpens et al., 2011; Malmer & Wallén, 2005), our study area has exceptionally low regional deposition (Harmens et al., 2015; Nickel et al., 2017), there are no point emission sources nearby, and therefore, we consider it unlikely that vegetation changes would considerably relate to nitrogen deposition in our case.

### 4.3 Autogenic or allogenic succession?

The question remains, whether the observed vegetation changes were driven by global change factors, such as climate warming. In the case of minerotrophic fens, disentangling the effects of climate change or local hydrological modifications from natural development is difficult, as they can have similar effects on fen vegetation (Tahvanainen, 2011). The autogenic peatland succession from rich fen to poor fen, and eventually to ombrotrophic, *Sphagnum*‐dominated bog is attributed to gradual isolation from minerogenic water due to long‐term peat accumulation and consequent vegetation change (Belyea & Clymo, 2001; Kuhry et al., 1993). In an allogenic process, this succession can be triggered by hydrological changes (Hughes, 2000; Hughes & Barber, 2004; Tahvanainen, 2011). For the most part, observed vegetation changes in our study area, indeed, follow typical bog development trajectory and can be considered to represent a progressive successional trend. Impact of gross‐scale autogenic development can be ruled out, as the water flow paths from catchment through the central parts of the mire area remain unaltered (Appendix S1). According to Kuznetsov et al. (2012), the long‐term peat growth rate was 0.75 mm/year, which is not nearly enough to affect hydrology in the 20‐year time span, considering the topographic relief of the mire area. Rich fen vegetation has prevailed in the study site since 8100 B. P., with alteration between *Scorpidium scorpioides* flarks and minerotrophic *Sphagnum* hummocks (Kuznetsov et al., 2012), that is, similar to spatial microsite variation of contemporary vegetation. Therefore, we look for allogenic factors that may have affected vegetation during recent decades.

In our study area, the 20‐year mean annual temperature rose from +0.9°C prior to our first sampling to +1.9°C prior to the second sampling. The effective temperature sum rose from 958 to 1093°C, respectively (Finnish Meteorological Institute, open data). This warming is continuation of a longer trend of recovery from the Little Ice Age (LIA), which persisted ca. 1300–1900 CE in the study area (Luoto & Nevalainen, 2017). From 1850 to 2020, mean temperature of Finland rose from +0.4°C to +2.8°C in a trend with recent acceleration after 1970s (Mikkonen et al. 2015). Development of wet aapa mire conditions has been connected to cool and wet phases of LIA (Arlen‐Pouliot & Payette, 2015), and many paleoecological studies have found post‐LIA increase of *Sphagnum* in northern fens (Loisel & Yu, 2013; Primeau & Garneau, 2021; Robitaille et al., 2021; van Bellen et al., 2018). Indeed, many of the changes in vegetation observed in our study were similar to what could be expected in northern fens in response to rising temperatures. On a global scale, the mean annual temperature is the most important factor to explain *Sphagnum* productivity (Gunnarsson, 2005). While *Sphagnum* moss cover can decline due to warming‐induced desiccation (Lyons et al., 2020; Norby et al., 2019), they can benefit from increased temperature and longer growing season, as long as moisture supply is not limited (Bengtsson et al., 2021; Küttim et al., 2020; Loisel et al., 2012). According to Zhao et al. (2017), *Sphagnum* mosses benefit also from decreasing snow cover, as they are less affected by frost than sedges. Of course, sensitivity of *Sphagnum* to climate is species‐specific, but, for example, *S*. *medium,* a generalist species that increased in our site, is able to take advantage of increasing temperature (Bengtsson et al., 2021; Küttim et al., 2020). In addition, rich fen hummock species *S. warnstorfii* benefits from the absence of high‐water events, making it competitive against rich fen brown mosses (Granath et al., 2010), which may explain its increase. Rich fen mosses, on the other hand, suffer from warmer winters with repeated thaw–freeze cycles (Küttim et al., 2019). Overall, our findings of greater changes in vegetation than in WTD or water chemistry suggests that global change factors have affected biotic responses and driven changes directly, rather than indirectly via changes of local environmental conditions. Our hydrological observations were limited to few sampling occasions, and it is possible that comparisons between datasets are affected by WT fluctuation. In general, fens have narrow fluctuation during growing season in Finland (Menberu et al., 2016; Tahvanainen et al., 2003). However, it is possible that even slight changes of water level or repeated drought events, unnoticed in our survey, and may have launched irreversible successional changes (Breeuwer et al., 2009; Granath et al., 2010).

Climate warming is predicted to cause falling of water‐table levels (WTL) in northern mires, particularly in areas with reduced precipitation (Gong et al., 2012). Falling WTL could alter plant community structure and ecosystem functions, such as CO_2_ exchange, as demonstrated by experiments with manipulations of WTL (Kokkonen et al., 2019; Laine et al., 2019; Mäkiranta et al., 2018). Overall, it is important to differentiate between a regressive process of falling WTL and a progressive process of increased growth, while both could eventually result in similarly increased WTD. In our study area, annual precipitation had increased, and our observations indicated that the WTL had not changed significantly. Thus, increased precipitation apparently had balanced out moisture loss caused by increased evaporation (Gong et al., 2012). The fact that hummock vegetation had still increased reflects, in our interpretation, a progressive response and accelerated succession with the natural trajectory of bog development (Belyea & Clymo, 2001). This is supported by the increased mismatch between vegetation and WTD, as it could reflect ongoing changes in plant communities.

In northern mires, many studies have predicted decreasing carbon sink capacity (Ise et al. 2008; Chaudhary et al., 2020; Hugelius et al., 2020), while some studies indicate sustained sink function in the future (Chaudhary et al., 2017; Spahni et al., 2013), and others have observed increase of peat accumulation (Charman et al., 2013; Loisel & Yu, 2013). Differing results are partly due to different methodological approaches and climate trajectories, but carbon fluxes differ significantly also between vegetation types (Turetsky et al., 2014). Vegetation is the key ecosystem structure affecting carbon cycle (Straková et al., 2012), and carbon accumulation rate differs, for example, between *Sphagnum*, non‐*Sphagnum*, and permafrost peat (Loisel et al., 2014; Treat et al., 2016). Therefore, a shift in vegetation composition can significantly alter carbon cycling (Gavazov et al., 2018; Straková et al., 2012), and decadal‐scale vegetation changes should be strongly considered in estimating future carbon sink capacity of northern mires.

## 5 CONCLUSIONS

Our study provided a unique case of repeated sampling of vegetation, water chemistry, and WTD in a pristine boreal fen after 20 years of the first fieldwork, a period with a marked shift to warmer climate conditions. Our results suggest that pristine boreal fens are not safe from global changes, and substantial vegetation changes are observable in just two decades. In our study area, observed vegetation changes were clearest in the rich fen, where *Sphagnum* mosses had increased at the expense of rich fen bryophytes, with little change in water chemistry gradient. Hummock vegetation increased over wet fen vegetation, while measurements of WTD did not show consistent drying. Instead, changes of WTD showed spatial variation and reflected vegetation dynamics. For the most part, vegetation changes conformed to typical pattern of natural succession, but recent warming has possibly amplified the process. The possibility of such progressive changes taking place in northern mires calls for more attention in research of ecosystem responses to global change.

## CONFLICT OF INTEREST

None declared.

## AUTHOR CONTRIBUTIONS

**Tiina H. M. Kolari:** Formal analysis (equal); funding acquisition (supporting); investigation (lead); methodology (equal); visualization (equal); writing–original draft (lead). **Pasi Korpelainen:** Investigation (supporting); methodology (supporting); visualization (equal). **Timo Kumpula:** Methodology (supporting); supervision (supporting); visualization (supporting); writing–review and editing (supporting). **Teemu Tahvanainen:** Formal analysis (equal); funding acquisition (lead); investigation (supporting); methodology (equal); supervision (lead); writing–review and editing (lead).

## Supporting information

Appendix S1

Appendix S2

## Data Availability

Total covers of bryophyte groups, plot designations to EUNIS habitat types, water‐table depths, pH, and base cations: Zenodo https://doi.org/10.5281/zenodo.4662571. Whole species and water chemistry data: Zenodo https://doi.org/10.5281/zenodo.4663755.
