# Supplementary material for: Accelerated vegetation succession but no hydrological change in a boreal fen during 20 years of recent climate change
Source: Ecol Evol. 2021 May 2;11(12):7602–21. doi: 10.1002/ece3.7592 (PMC8216969; doi:10.1002/ece3.7592)
Supplement: Supplementary file 1 — Appendix S1 [file ECE3-11-7602-s001.pdf]

Supplement 1

Accelerated vegetation succession but no hydrological change in a boreal fen during 20 years of recent climate change

Tiina H. M. Kolari, Pasi Korpelainen, Timo Kumpula & Teemu Tahvanainen

Additional results on spatial and temporal variation in water chemistry

1. Catchment and flow-path analysis

2. Spatial interpolations of water chemical variation

3. Temporal variation of water-table depth and water chemistry at four monitoring sites

4. Correlations of water chemistry variables

1. Catchment and flow-path analysis

We used a LIDAR-based, 2-m resolution digital elevation model (DEM), produced by the National Land Survey of Finland, to produce delineations of catchment and water flowpaths in the 'Härkösuo' mire. The methodology is explained in more detail by Sallinen et al. (2019) and general interpretation of water flow directions in relation to vegetation and water chemistry patterns is discussed by Tahvanainen et al (2002).

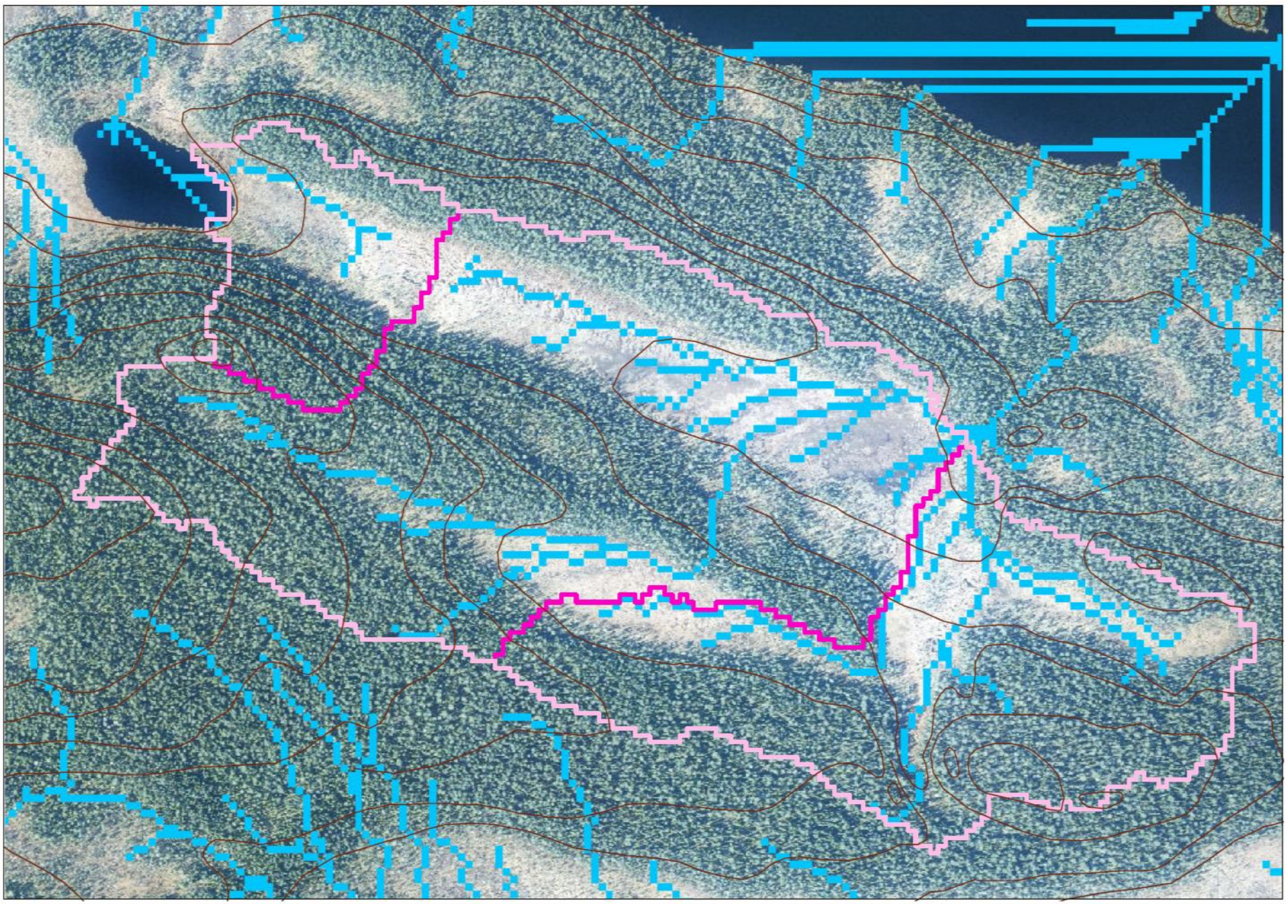

The pink line delineates the catchment of the whole mire basin and purple lines indicate sub-catchment divides. Catchment relief is indicated with 2.5-m contours. The blue lines indicate water flowpaths, i.e. main routes and directions of water flow, across the mire area. Water flows from the mire area to the lake Palonen in NE corner of the figure.

2. Spatial interpolations of water chemical variation

This section has Ordinary Kriging interpolation maps of water chemical variables over the sampled mire area. In all four-panel figures, interpolations of grid-point data (n = 48) are presented for 1998 and 2018 and a separate map is presented of differences between the two data sets. In addition, interpolation maps are presented of all data points in 2018 (n = 195). Thus, the grid interpolations are directly comparable between 1998 and 2018, while the all-points interpolations can be compared to the grid-point interpolations of 2018 data. In our interpretation, the grid-point data show closely similar patterns, as compared to the all-point interpolations.

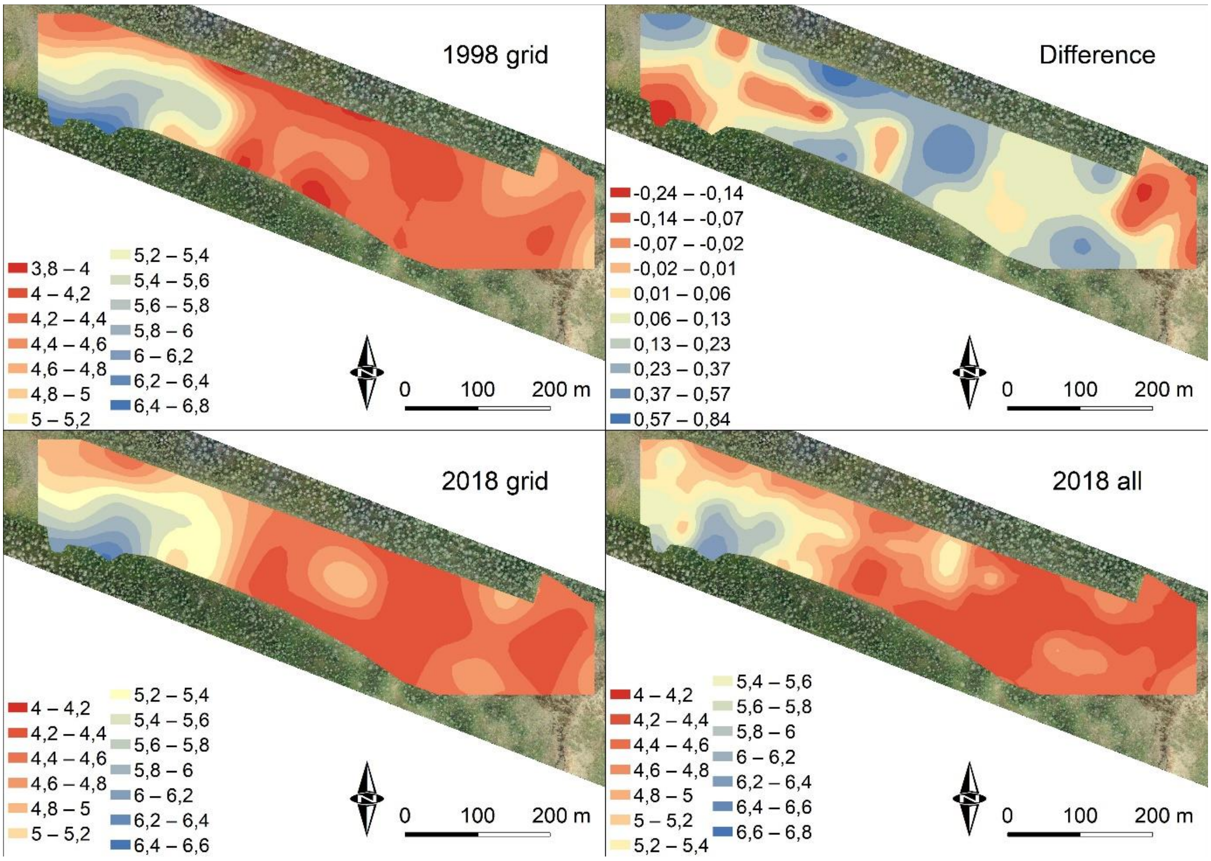

Spatial variation of water pH.

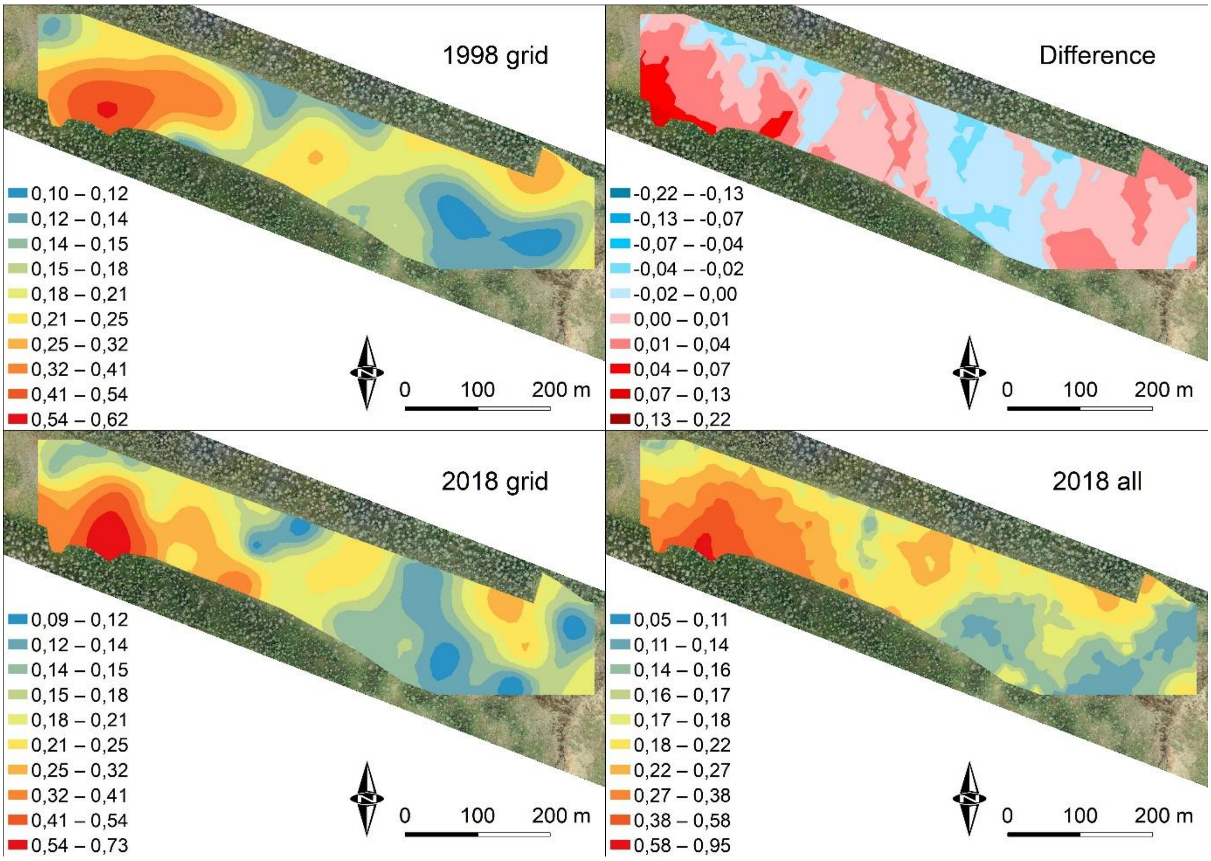

Spatial variation of water Base cations (meq/l).

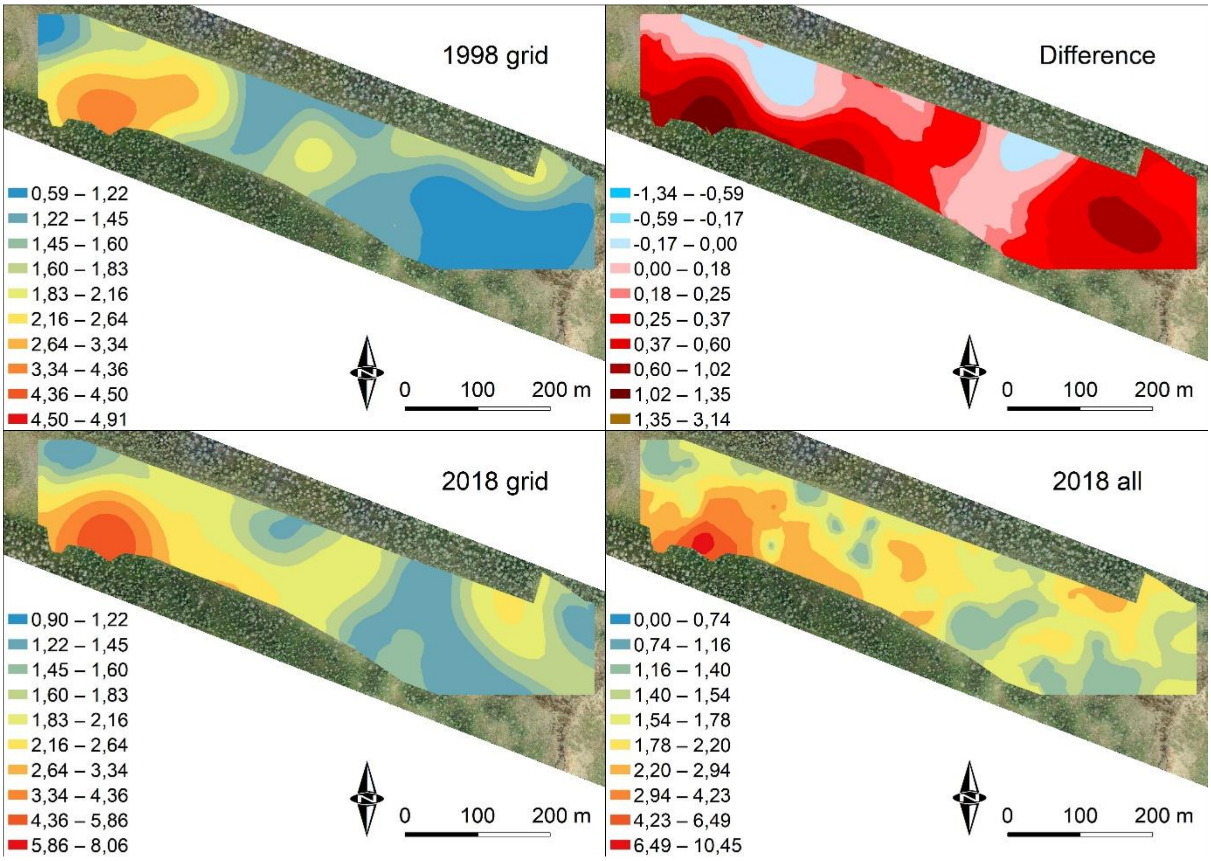

Spatial variation of water Ca (mg/l).

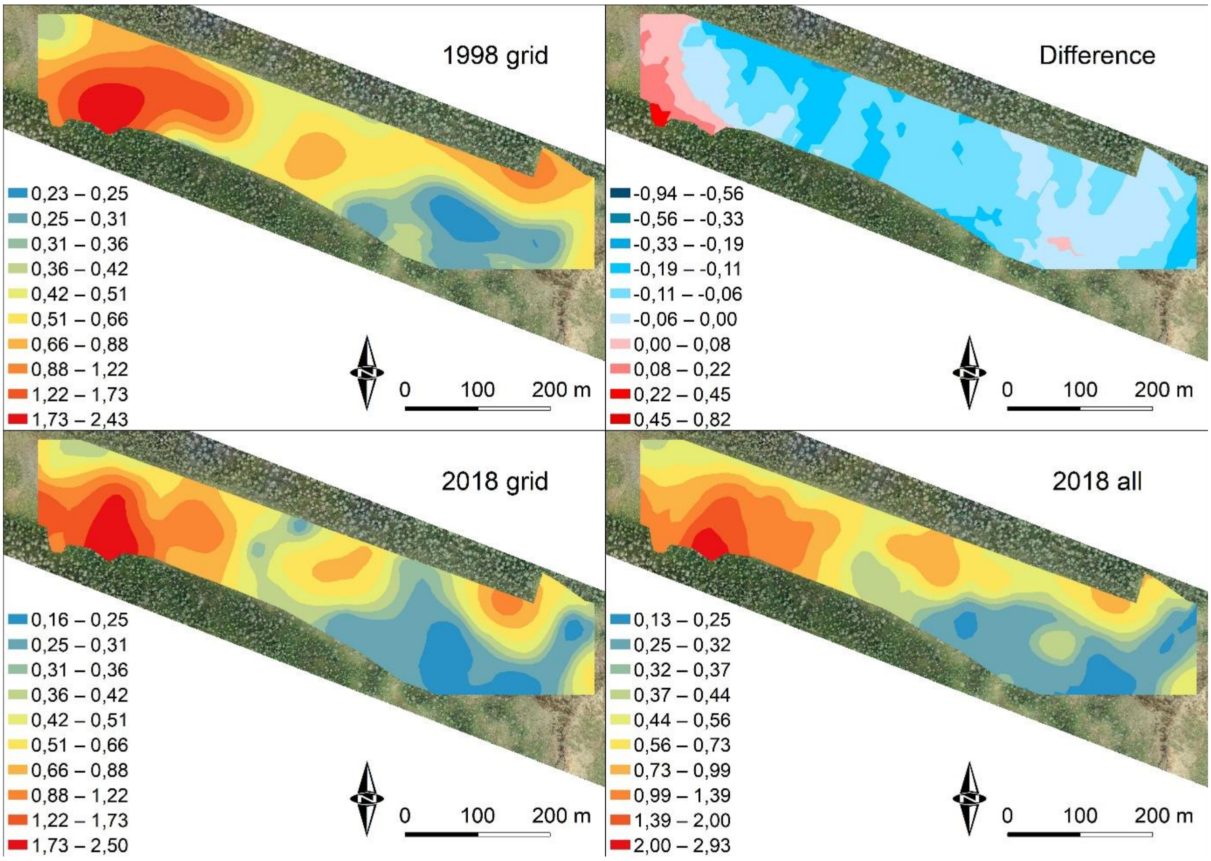

Spatial variation of water Mg (mg/l).

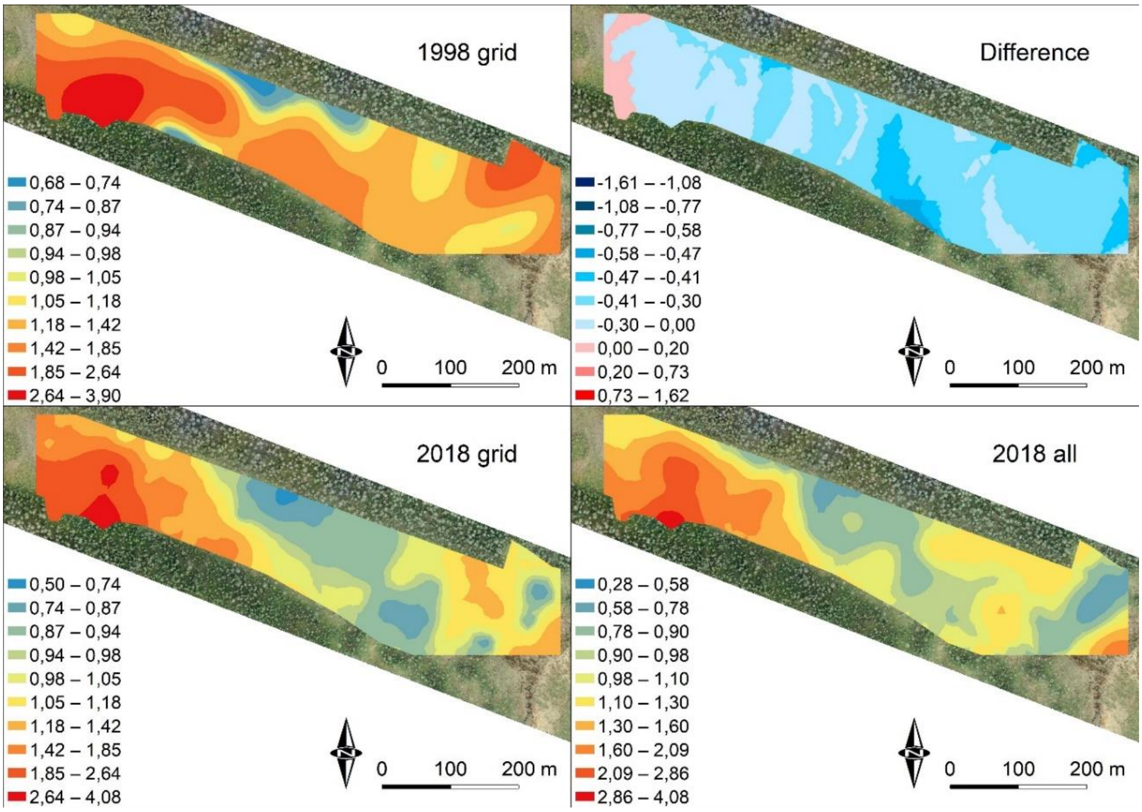

Spatial variation of water Na (mg/l).

3. Temporal variation of water-table depth and water chemistry at four monitoring sites

Background

In the article, we mainly focus our comparison of water chemical patterns on two data sets covering the whole mire area. These data sets are “snap-shots” of the late summer situations in 1998 and 2018. This makes it possible to compare spatial patterning and zonation related to vegetation. While the main interest lies on possible long-term changes, short-term variation can be equally significant and potentially mask longer-term trend. In this supplement we make an account on temporal variation of water chemistry.

Compilations of measurements from different occasions are shown for four sampling sites and all results are from years 1998, 1999, 2018, 2019 and 2020. The 1998 data are single observations from data of Tahvanainen et al (2002). The 1999 data are presented as minimum to maximum range and arithmetic averages of weekly monitoring (n = 16) during the growing season. This monitoring data was published in Tahvanainen et al (2003) except for the data of the open spring (HLA01), which is also included here. In 2018, 2019 and 2020 we collected few samples in addition to the 15th August 2018 main sampling that are shown here. Notice that the dates are not the same for all sites. The additional sampling was conducted in dates 29th May, 7th June, 20th September 2018, 15th August 2019, and 10th August 2020.

Key to figure symbols:

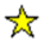

August 1998, data from Tahvanainen et al. (2002).

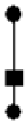

Seasonal minimum, mean and maximum from 1999, data from Tahvanainen et al (2003).

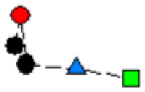

Observations 2018-2020, red circle 15th August 2018, blue triangle 15th August 2019, green square 10th August 2020.

All element concentrations are presented as mg/l. We have selected elements that have concentrations above detection limits in all data sets, that are not potentially affected by contamination (comparison with control samples of ultra-filtered deionized water) and that are potentially important to plants.

The water-table measurements are presented as water-table depth relative to moss surface, i.e. measurements are conducted from pipe wells and an approximate value of pipe length extending from moss surface is extracted. At the rich fen site (H300), water-table was measured relative to a wooden reference pole that was inserted and cut to the approximate (floating) moss surface in 1998, and remained firmly fixed in peat through the study period.

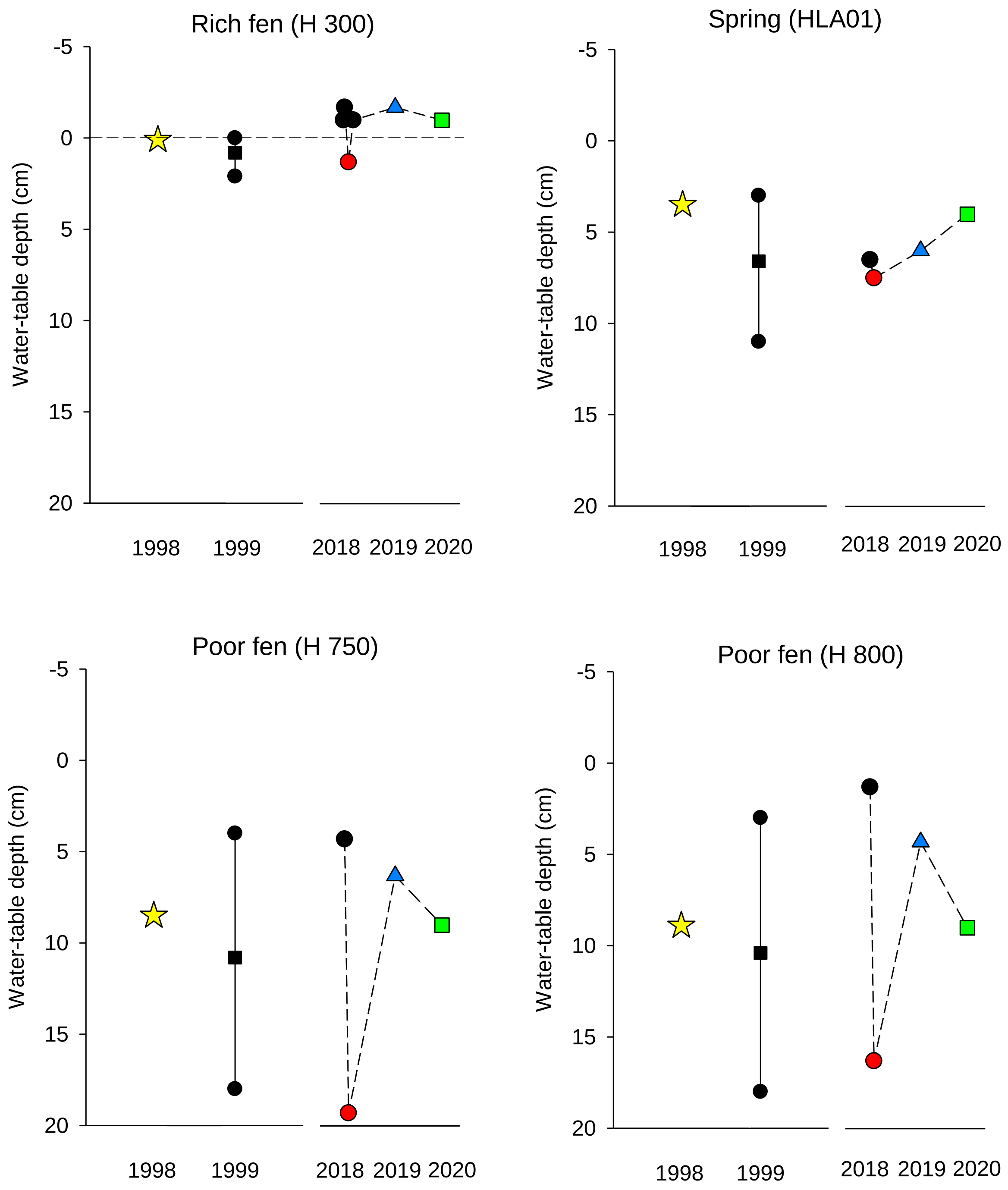

* fixed reference level is marked with hatched line for Rich fen (H300)

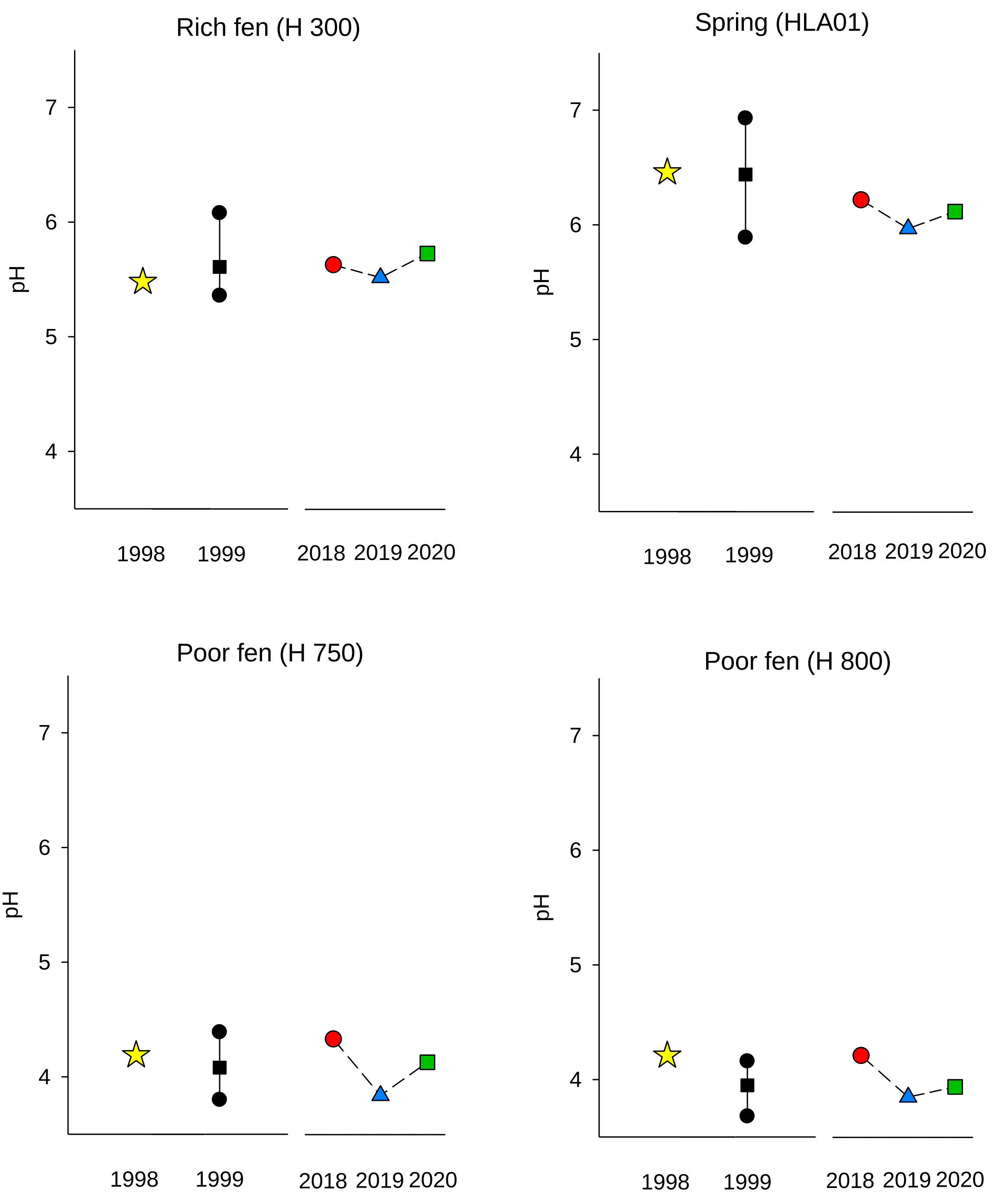
Rich fen (H 300)
Spring (HLA01)
Poor fen (H 750)
Poor fen (H 800)
pH
7
6
5
4
1998
1999
2018
2019
2020

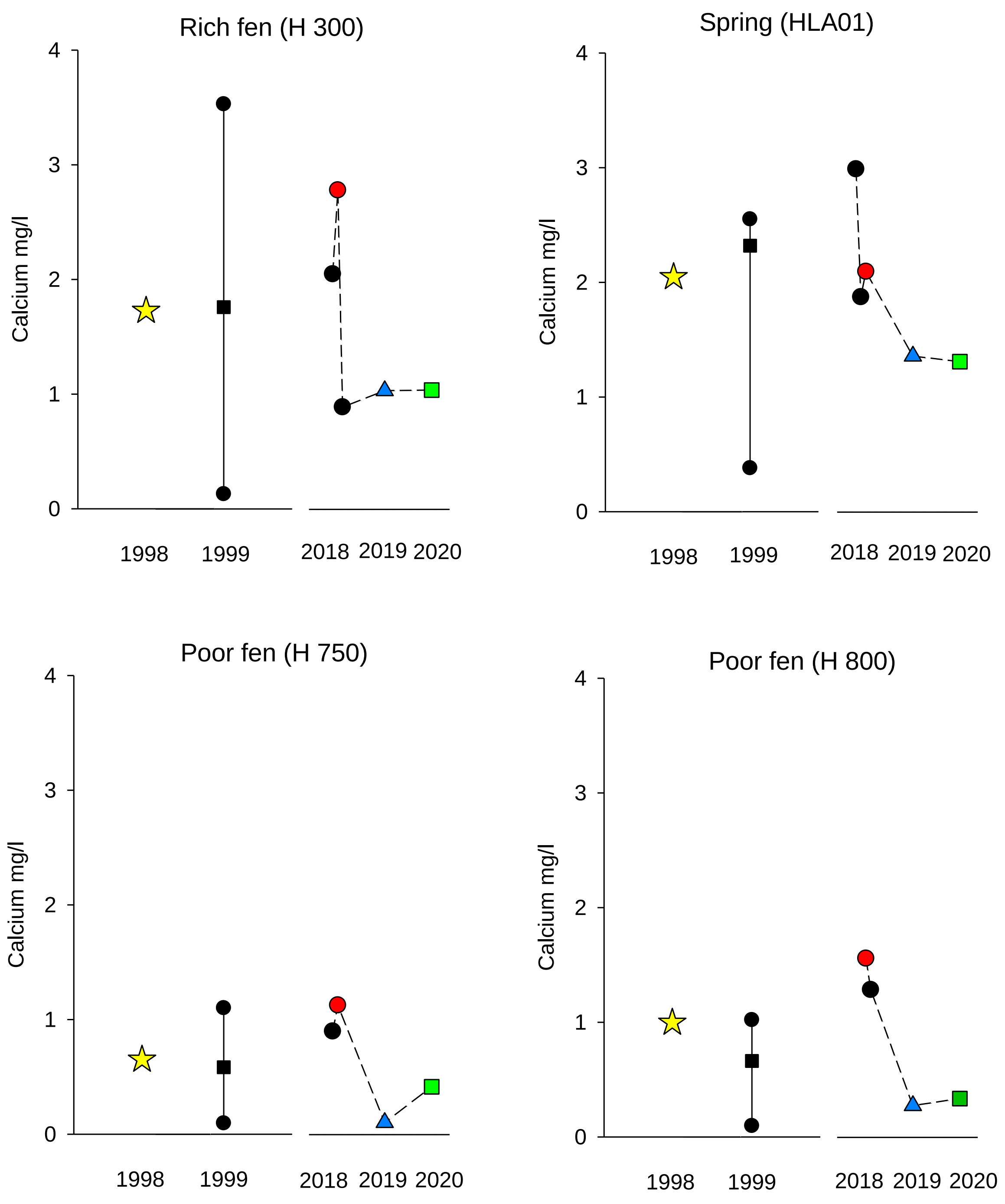
Rich fen (H 300)
Spring (HLA01)
Poor fen (H 750)
Poor fen (H 800)
Calcium mg/l
0
1
2
3
4
1998
1999
2018
2019
2020

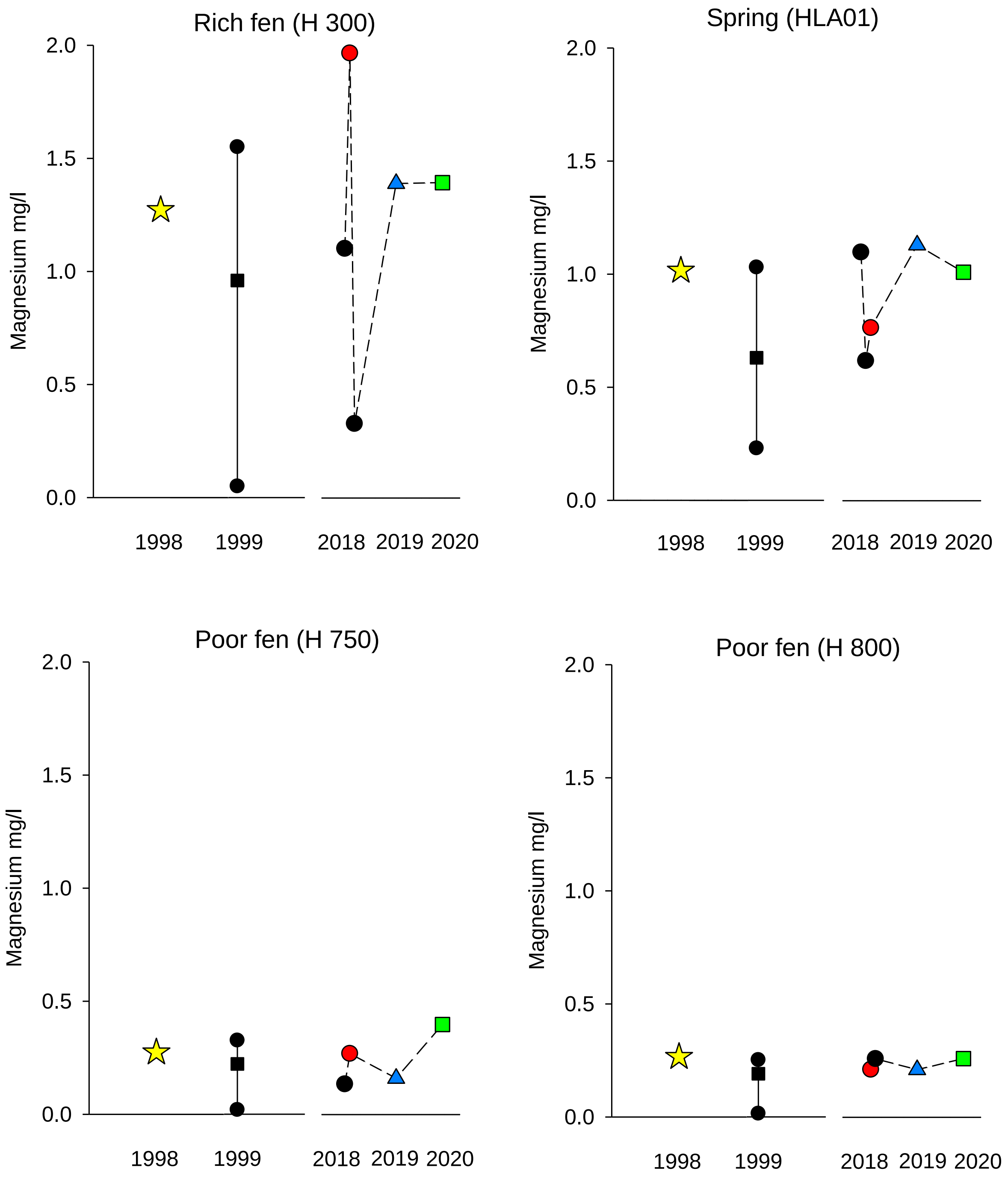
Rich fen (H 300)
Spring (HLA01)
Poor fen (H 750)
Poor fen (H 800)
Magnesium mg/l
2.0
1.5
1.0
0.5
0.0
1998
1999
2018
2019
2020

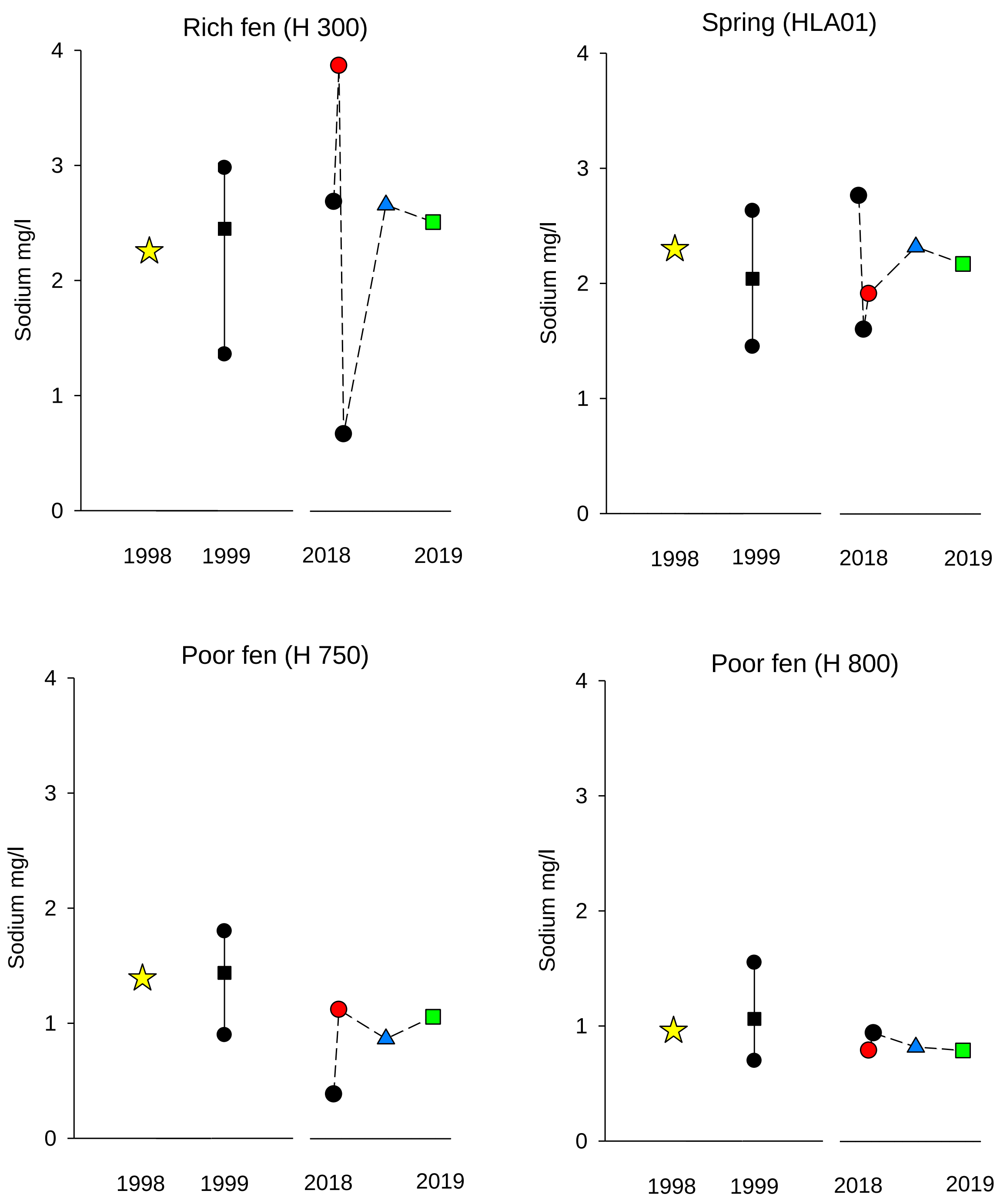
Rich fen (H 300)
Spring (HLA01)
Poor fen (H 750)
Poor fen (H 800)
Sodium mg/l
0
1
2
3
4
1998
1999
2018
2019

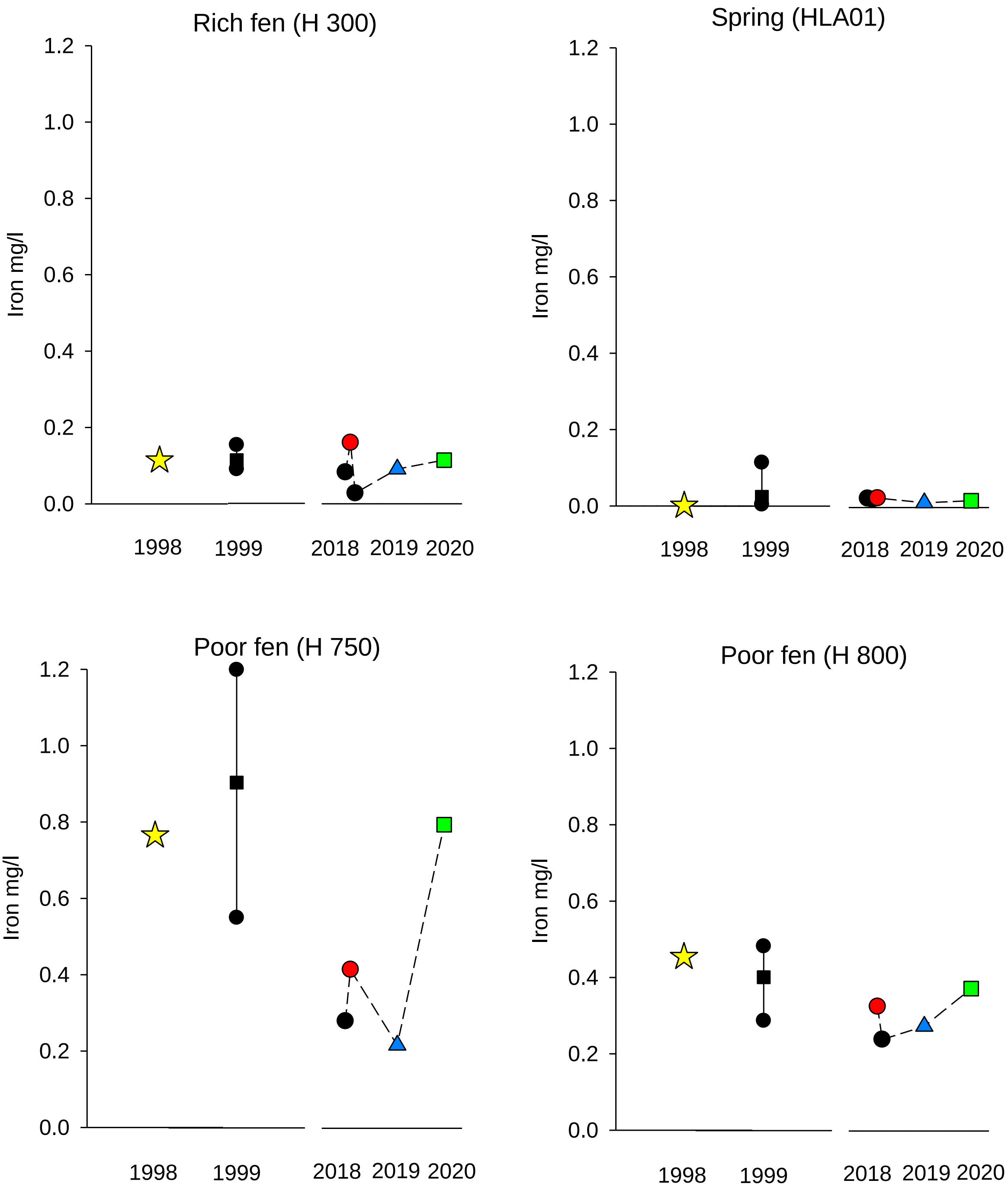
Rich fen (H 300)
Spring (HLA01)
Poor fen (H 750)
Poor fen (H 800)
Iron mg/l
1.2
1.0
0.8
0.6
0.4
0.2
0.0
1998
1999
2018
2019
2020

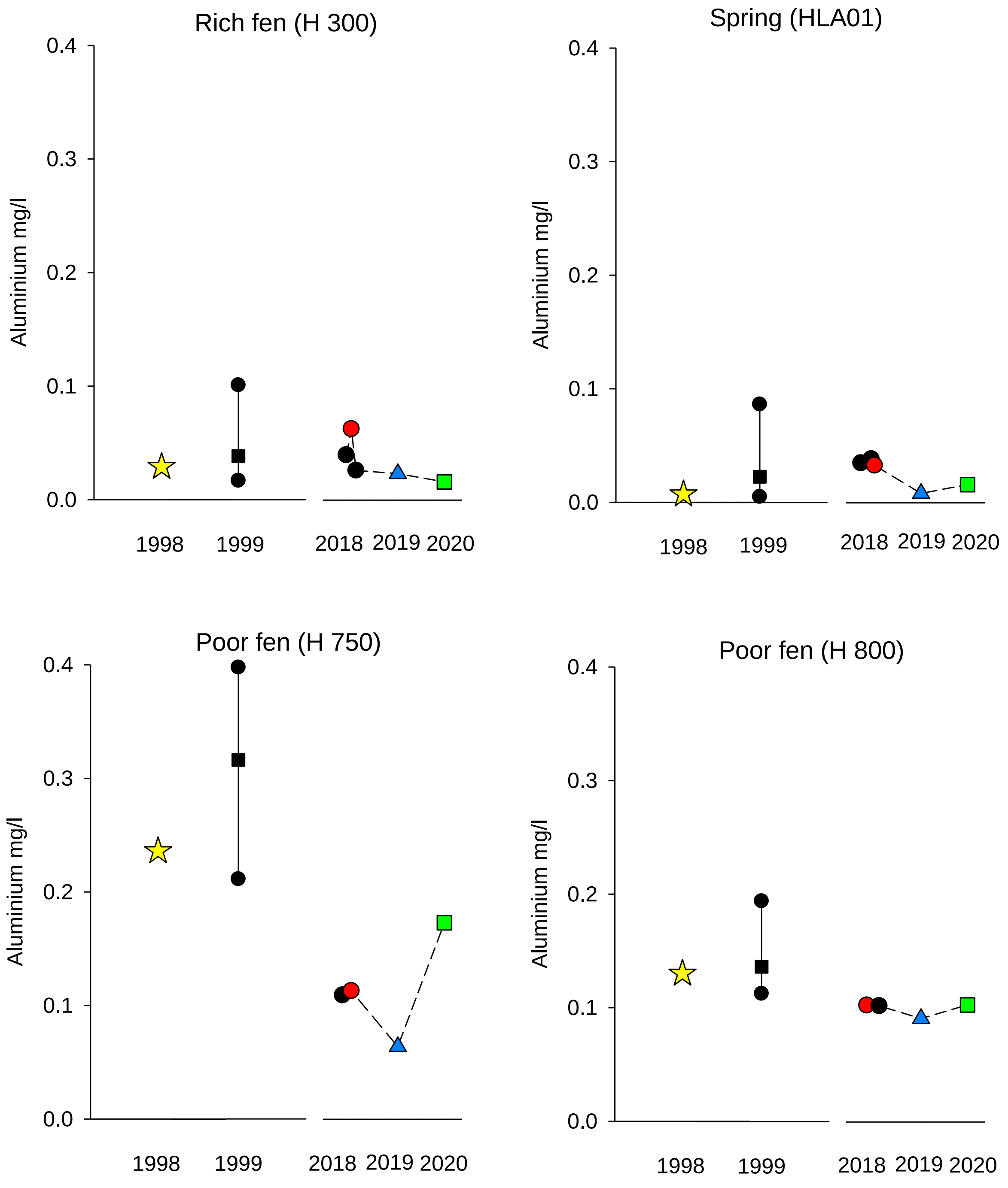
Rich fen (H 300)
Spring (HLA01)
Poor fen (H 750)
Poor fen (H 800)
Aluminium mg/l
0.0
0.1
0.2
0.3
0.4
1998
1999
2018
2019
2020

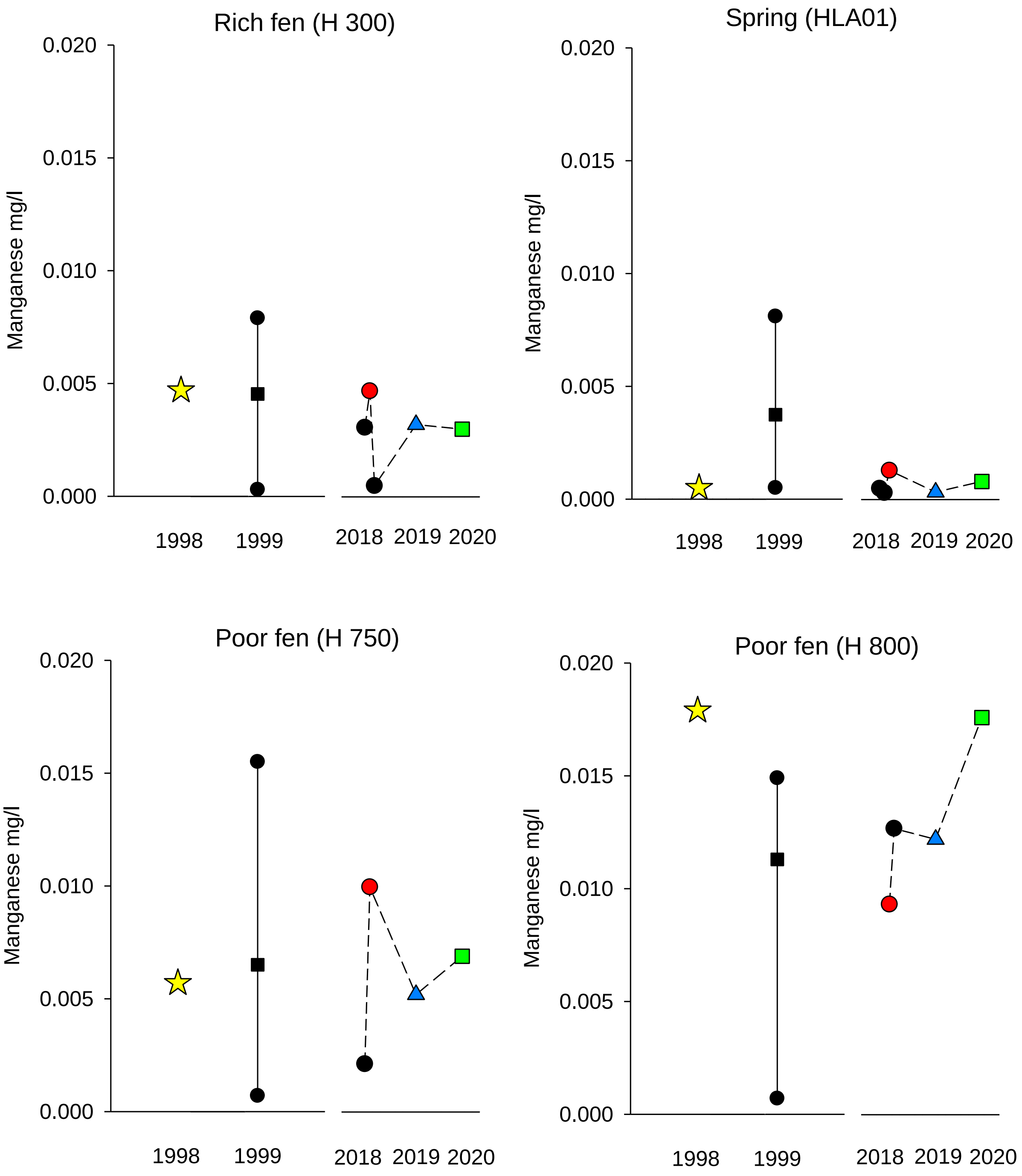

Rich fen (H 300)
Spring (HLA01)
Poor fen (H 750)
Poor fen (H 800)
Manganese mg/l
0.020
0.015
0.010
0.005
0.000
1998
1999
2018
2019
2020

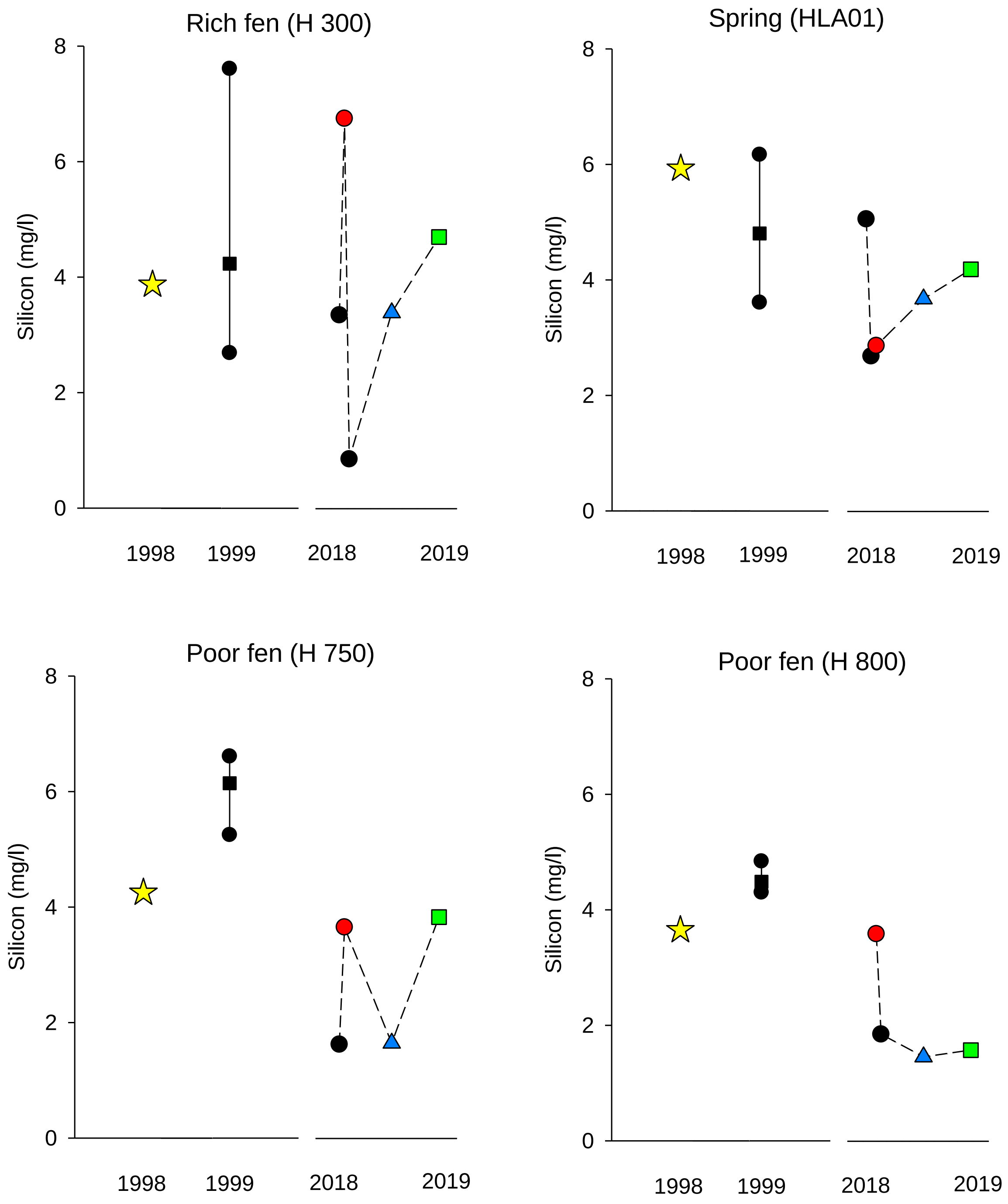
Rich fen (H 300)
Spring (HLA01)
Poor fen (H 750)
Poor fen (H 800)
Silicon (mg/l)
0
2
4
6
8
1998
1999
2018
2019

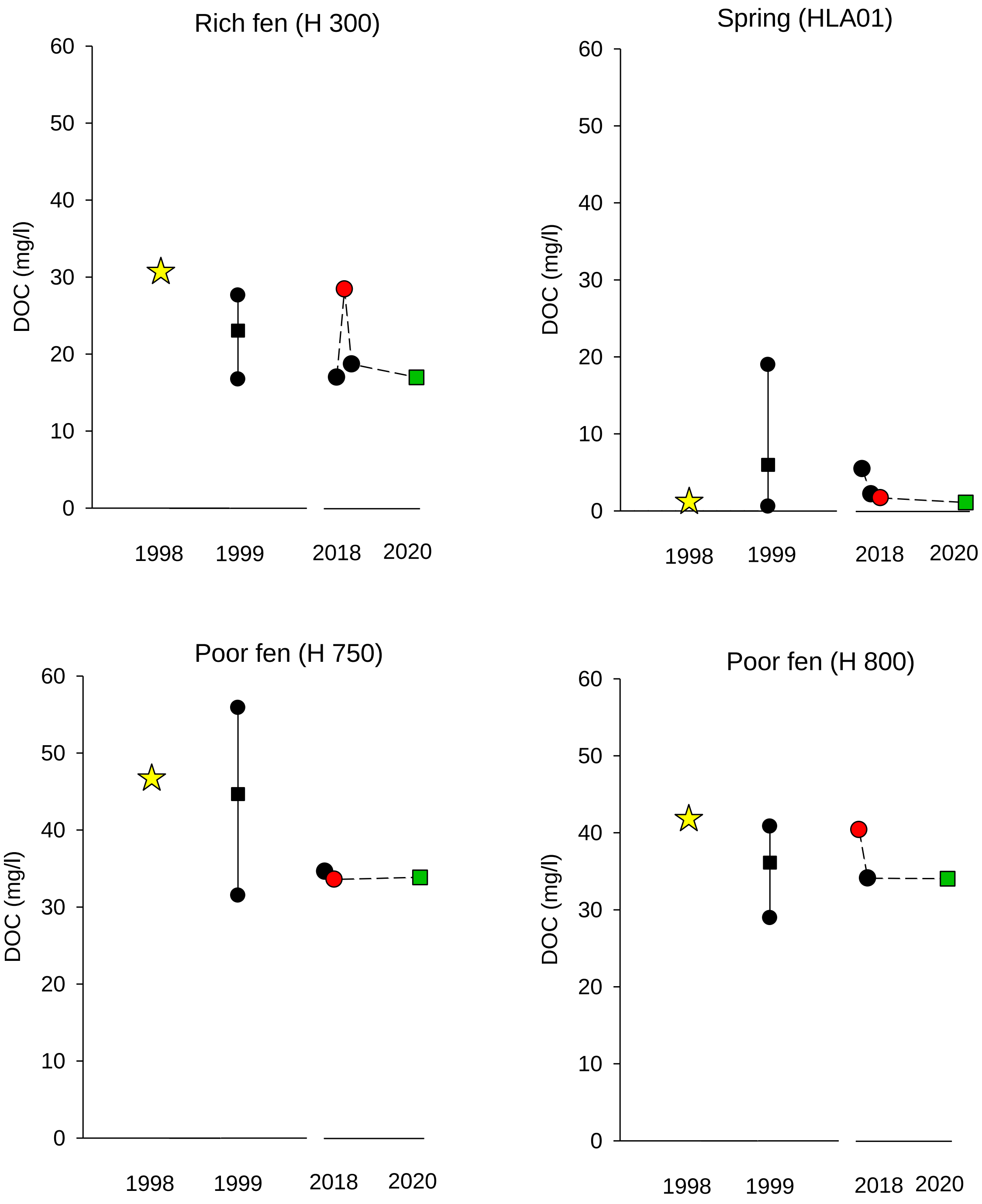

Rich fen (H 300)
Spring (HLA01)
Poor fen (H 750)
Poor fen (H 800)
DOC (mg/l)
0
10
20
30
40
50
60
1998
1999
2018
2020

4. Correlations of water chemistry variables

All correlation coefficients presented here are parametric Pearson correlations, thus, subject to assumption of normal distribution. We explored also non-parametric correlations and checked that all reported strong (r > 0.5) and significant (p < 0.01) correlations (bold font) were also significant as Spearman rank correlations.

Table 1. Pearson correlation results for water chemistry variables (n = 195) and poor-rich index (n = 126) in the 2018 data. Similarly as in the 1998 data (Tahvanainen et al. 2002), pH had the strongest correlation with Mg, Na, and the sum of equivalent charges of main base cations (Ca, Mg, Na). The Poor-rich index was calculated for each vegetation plot as the difference between total cover of rich fen Bryidae and total cover of poor fen *Sphagnum* species, as in Tahvanainen et al. (2002). Plots with zero poor-rich index value were omitted from the correlation analysis. Poor-rich index had the strongest correlation with pH. In 1998 data, poor-rich index correlated strongly with pH (r = 0.82), Mg (r = 0.75), and Na (r = 0.70), while in our 2018 data, all of these correlations were clearly weaker.

	Mean	SD	pH	DOC (mg/l)	Al (mg/l)	Ca (mg/l)	Fe (mg/l)	Mg (mg/l)	Mn (mg/l)	Na (mg/l)	Si (mg/l)	Base cations
pH	5.04	0.65	-									
DOC (mg/l)	29.3	12.1	**-0.68**									
Al (mg/l)	0.09	0.08	**-0.58**	**0.65**								
Ca (mg/l)	2.35	1.70	**0.56**	-0.36	-0.14							
Fe (mg/l)	0.29	0.30	-0.49	**0.67**	**0.78**	-0.09						
Mg (mg/l)	0.83	0.64	**0.77**	-0.47	-0.39	**0.86**	-0.26					
Mn (mg/l)	0.01	0.02	-0.02	0.16	0.11	0.14	0.21	0.14				
Na (mg/l)	1.44	0.79	**0.68**	-0.45	-0.26	**0.72**	-0.18	**0.86**	0.08			
Si (mg/l)	2.79	1.55	-0.14	0.14	0.49	0.16	0.54	0.08	0.12	0.30		
Base cations (meq/l)	0.27	0.17	**0.68**	-0.43	-0.25	**0.94**	-0.17	**0.96**	0.14	**0.89**	0.18	
Poor-rich index	-3.4	46.8	**0.64**	**-0.52**	**-0.57**	0.26	-0.58	**0.50**	-0.12	0.42	-0.33	0.38

Table 2. Pearson correlations for water chemistry variables between the 1998 and 2018 data (n = 48).

		**2018** pH	DOC	Al (mg/L)	Ca (mg/L)	Fe (mg/L)	Mg (mg/L)	Mn (mg/L)	Na (mg/L)	Si (mg/L)
**1998**	pH	**0.93**	**-0.66**	-0.48	**0.66**	-0.35	**0.85**	-0.27	**0.76**	0.17
	DOC	**-0.75**	**0.68**	**0.53**	-0.48	**0.52**	**-0.61**	0.21	**-0.53**	0.01
	Al (mg/L)	-0.38	**0.60**	**0.81**	-0.01	**0.56**	-0.29	0.26	-0.15	0.22
	Ca (mg/L)	**0.69**	-0.32	-0.28	**0.76**	-0.25	**0.75**	-0.26	**0.55**	-0.06
	Fe (mg/L)	-0.36	**0.56**	**0.60**	-0.14	**0.70**	-0.27	0.19	-0.19	0.26
	Mg (mg/L)	**0.78**	-0.44	-0.38	**0.76**	-0.35	**0.83**	-0.32	**0.64**	-0.03
	Mn (mg/L)	-0.28	0.33	0.49	0.05	0.45	-0.17	0.42	-0.09	0.17
	Na (mg/L)	**0.70**	**-0.53**	-0.28	**0.70**	-0.27	**0.75**	-0.22	**0.68**	0.15
	Si (mg/L)	0.26	-0.12	0.20	0.26	0.15	0.29	0.01	0.33	**0.51**
