# Supplementary material for: Accelerated vegetation succession but no hydrological change in a boreal fen during 20 years of recent climate change
Source: Ecol Evol. 2021 May 2;11(12):7602–21. doi: 10.1002/ece3.7592 (PMC8216969; doi:10.1002/ece3.7592)
Supplement: Supplementary file 2 — Appendix S2 [file ECE3-11-7602-s002.pdf]

**Supplement 2**

Accelerated vegetation succession but no hydrological change in a boreal fen during 20 years of recent climate change

Tiina H. M. Kolari, Pasi Korpelainen, Timo Kumpula & Teemu Tahvanainen

## Additional results on vegetation changes

1. Plant species autecology in Eastern Finland assessed with external data and literature.

2. Summary of results from SumF-tests

3. Summary of results from Blocked Indicator Species Analysis (bISA)

## 1. Plant species autecology in Eastern Finland assessed with external data and literature

A catalogue of plant species autecology is presented with weighted averages and standard deviations of water-table depth and water pH, and a niche width index. All data used here is from Eastern Finland, east from ca. 27° 37', and between ca. 62° 13' and 66° 30', collected from ca. 50 separate natural mire areas. The bulk of the data are visual cover estimates from 0.25 $m^2$ vegetation plots. Water-table depth was measured from perforated pipe wells and water pH measurements were performed from samples collected from the pipe wells. Since some species were absent from the plot data (marked with asterisk *), they were assessed by relevé data from spring fens (Ringa Luostarinen, unpubl.). These relevés are larger (mainly 2 to 16 $m^2$), water samples for pH were collected directly from open water and water-table depth measurements were not available.

Weighted averages (WA) and standard deviations (WSD) were calculated for WTD and pH using species abundance values (% cover) as weights. The WA values indicate center and WSD values the width of species distribution pattern along the environmental gradients. The "mire niche width" was calculated using information of the species autecological table of Eurola et al. (2015). Eurola et al. (2015) listed mire species' occurrences in different categories of mire sites. Within these categories we gave different classes weights according to the indication of that class concerning niche width. These weights are given in brackets in the following list of mire site categories.

1) The trophic gradient categories of Finnish mire site type classification with four classes: ombrotrophic (3), oligotrophic (3), mesotrophic (2), and eutrophic (1). These categories grossly interpret to bog, poor fen, intermediate fen, and rich fen.

2) The microtopographic gradient with three classes: hummock (3), intermediate (2), and hollow (1).

3) The main mire site types with six classes: spring mires (1), alluvial swamps (2), swamp forests (2), bog forests (3), open mires (2), and rich fens (1).

The mire niche width index was calculated as the weighted sum of occurrence in different category classes, divided by maximum sum of occurrences in the classes. In short, a value '1' would mean that a species would be present in all trophic and microtopographic classes of all main mire site types. The minimum score is '0.12' for a species that would be present only in hollows of eutrophic rich fens or spring fens. The different weights for different classes were necessary for variety of reasons. The species pools is cumulative across the trophic gradient towards eutrophic habitats, i.e. most ombrotrophic species also occur in oligotrophic to mesotrophic habitats, oligotrophic species extend to mesotrophic and eutrophic habitats, and all mesotrophic species thrive in eutrophic habitats, while by definition of the gradient, there is no extended occurrence of species in the other direction. In the microtopographic

gradient, there is wide range in heights of hummocks (some 20 to 60 cm), while the wet end of the gradient is narrow (hollows commonly defined as less than 5 cm WTD). In the main mire site types, rich fens and spring fens are most strictly defined by occurrence of specific indicator species.

According to the median mire niche width, species are indicated either as generalists (G) or specialists (s) in the column for mire niche group. In addition, many species are not strict mire plants, in fact nearly all plant species can be found also in other habitats in some regions. We have indicated occurrence in other habitats if that habitat rather than mires was the primary habitat of the species in the study area (F = forest habitats, A = aquatic habitats, M = meadows and grassland habitats).

| | Ext.data n | WTD WA | WTD WSD | pH WA | pH WSD | Mire niche width | Mire niche group | Primary habitat |
|---|---|---|---|---|---|---|---|---|
| **Bryophytes** | | | | | | | | |
| *Aulacomnium palustre* | 51 | 17.4 | 10.5 | 6.0 | 0.4 | 0.58 | G | |
| *Bryum weigelii* | 13* | | | 6.3 | 0.3 | 0.17 | s | |
| *Straminergon stramineum* | 29 | 6.7 | 5.6 | 5.1 | 0.7 | 0.50 | G | |
| *Calliergon cordifolium* | 12 | | | 6.2 | 0.0 | 0.21 | s | |
| *Calliergonella cuspidata* | 1 | 4.0 | | 6.4 | 0.2 | 0.35 | s | |
| *Campylium stellatum* | 74 | 6.2 | 4.6 | 6.4 | 0.2 | 0.13 | s | |
| *Cinclidium subrotundum* | 2 | 11.3 | 5.7 | 5.7 | 0.2 | 0.27 | s | |
| *Cinclidium stygium* | 15 | 5.1 | 2.7 | 6.1 | 0.4 | 0.15 | s | |
| *Dicranum angustum* | 3 | 11.0 | 1.8 | 5.9 | 0.2 | 0.33 | s | |
| *Dicranum bergenii* | 2 | 18.2 | 18.3 | 5.3 | 1.8 | 0.46 | G | |
| *Dicranum bonjeanii* | 18 | 12.2 | 9.4 | 6.3 | 0.4 | 0.27 | s | |
| *Helodium blandowii* | 4 | 5.8 | 1.5 | 6.9 | 0.1 | 0.29 | s | |
| *Hylocomiastrum umbratum* | 3* | | | 5.8 | 0.8 | 0.31 | s | |
| *Loeskypnum badium* | 37 | 7.2 | 6.4 | 5.9 | 0.6 | 0.38 | G | |
| *Paludella squarrosa* | 27 | 6.0 | 2.7 | 6.7 | 0.3 | 0.27 | s | |
| *Philonotis fontana* | 2 | 4.0 | 0.0 | 6.4 | 0.0 | 0.19 | s | |
| *Plagiomnium ellipticum* | 4 | 5.1 | 1.1 | 6.8 | 0.3 | 0.35 | s | |
| *Pleurozium schreberi* | 37 | 17.1 | 5.8 | 6.3 | 0.4 | 0.46 | G | F |
| *Polytrichum commune* | 2 | 10.1 | 0.7 | 6.6 | 0.1 | 0.46 | G | F |
| *Polytrichum strictum* | 12 | 23.2 | 12.7 | 6.1 | 0.4 | 0.46 | G | |
| *Pseudo-calliergon trifarium* | 7 | 1.5 | 4.1 | 5.8 | 0.4 | 0.19 | s | |
| *Rhizomnium magnifolium* | 35* | | | 6.1 | 0.3 | 0.17 | s | |
| *Rhizomnium pseudopunctatum* | 7 | 12.1 | 10.0 | 6.3 | 0.1 | 0.29 | s | |
| *Rhizomnium punctatum* | 14* | | | 5.9 | 0.3 | 0.31 | s | |
| *Rhytidiadelphus triquetrus* | 7* | | | 6.0 | 0.2 | 0.27 | s | F |
| *Sarmentypnum sarmentosum* | 7 | 7.1 | 7.1 | 5.5 | 0.3 | 0.25 | s | |
| *Scorpidium scorpioides* | 11 | 3.1 | 3.1 | 6.3 | 0.2 | 0.23 | s | |
| *Scorpidium revolvens* | 14 | 5.4 | 4.1 | 6.1 | 0.3 | 0.12 | s | |
| *Sphagnum recurvum agg.* | 64 | 13.1 | 4.9 | 4.8 | 0.9 | 0.65 | G | |
| *S. annulatum* | 7 | 9.5 | 4.7 | 4.6 | 0.4 | 0.27 | s | |
| *S. aongstroemii* | 1 | 18.0 | | 5.1 | | 0.40 | G | |
| *S. capillifolium* | 1 | 45.0 | | 6.2 | | 0.46 | G | F |
| *S. compactum* | 4 | 5.0 | 3.0 | 3.9 | 0.4 | 0.29 | s | |
| *S. contorum* | 6 | 11.0 | 3.3 | 6.3 | 0.4 | 0.31 | s | |
| *S. balticum* | 16 | 9.9 | 4.8 | 4.0 | 0.1 | 0.42 | G | |
| *S. fuscum* | 58 | 15.8 | 7.2 | 5.2 | 1.0 | 0.46 | G | |

| | | | | | | | | |
|---|---|---|---|---|---|---|---|---|
| *S. jensenii* | 15 | 6.3 | 2.5 | 4.4 | 0.3 | 0.35 | s | |
| *S. lindbergii* | 6 | 6.1 | 0.5 | 4.1 | 0.1 | 0.38 | G | |
| *S. cf. magellanicum* | 27 | 9.9 | 4.8 | 4.9 | 0.5 | 0.62 | G | |
| *S. majus* | 17 | 4.2 | 2.9 | 4.1 | 0.3 | 0.29 | s | |
| *S. papillosum* | 25 | 10.6 | 4.5 | 5.4 | 0.9 | 0.37 | G | |
| *S. platyphyllum* | 10 | 5.0 | 5.5 | 4.7 | 0.4 | 0.33 | s | A |
| *S. pulchrum* | 14 | 5.2 | 1.9 | 3.8 | 0.3 | 0.33 | s | |
| *S. riparium* | 15* | | | 5.5 | 0.7 | 0.29 | s | |
| *S. rubellum* | 12 | 15.7 | 3.8 | 5.1 | 1.0 | 0.46 | G | |
| *S. russowii* | 14 | 11.7 | 13.1 | 5.1 | 0.7 | 0.50 | G | |
| *S. subfulvum* | 20 | 12.3 | 9.9 | 5.9 | 0.6 | 0.31 | s | |
| *S. subsecundum* | 7 | 3.6 | 3.3 | 5.2 | 0.5 | 0.38 | G | |
| *S. tenellum* | 1 | 4.0 | | 4.3 | | 0.38 | G | |
| *S. teres* | 3 | 5.7 | 10.0 | 6.1 | 0.1 | 0.33 | s | |
| *S. warnstorfii* | 86 | 13.7 | 7.2 | 6.1 | 0.4 | 0.23 | s | |
| *Tomentypnum nitens* | 44 | 8.5 | 6.8 | 6.5 | 0.3 | 0.29 | s | |
| *Warnstorfia exannulata* | 3 | 8.9 | 1.5 | 5.5 | 0.8 | 0.27 | s | |
| *Warnstorfia fluitans* | 5 | 2.3 | 5.6 | 4.9 | 0.4 | 0.35 | s | |
| *Warnstorfia procera* | 5 | 7.7 | 6.6 | 5.2 | 0.5 | 0.27 | s | |
| *Aneura pinguis* | 36 | 11.1 | 7.5 | 6.3 | 0.3 | 0.27 | s | |
| *Lophozia rutheana* | 29 | 12.0 | 3.8 | 6.4 | 0.1 | 0.12 | s | |
| *Mylia anomala* | 54 | 16.6 | 8.0 | 5.6 | 1.1 | 0.54 | G | |
| *Scapania paludosa* | 2 | 8.5 | 2.0 | 5.4 | 0.7 | 0.15 | s | |
| **Vascular plants** | | | | | | | | |
| *Andromeda polifolia* | 159 | 12.0 | 7.0 | 5.0 | 1.0 | 0.50 | G | |
| *Angelica sylvestris* | 20 | 13.6 | 9.0 | 6.4 | 0.2 | 0.42 | G | F |
| *Betula nana* | 80 | 12.6 | 7.5 | 5.4 | 0.8 | 0.62 | G | |
| *Calluna vulgaris* | 33 | 18.4 | 8.7 | 5.9 | 1.0 | 0.46 | G | F |
| *Carex chordorrhiza* | 29 | 5.5 | 7.0 | 6.1 | 0.6 | 0.35 | s | |
| *Carex dioica* | 22 | 16.8 | 11.2 | 6.1 | 0.4 | 0.42 | G | |
| *Carex globularis* | 2 | 20.0 | 7.1 | 4.7 | 0.0 | 0.35 | s | |
| *Carex lasiocarpa* | 105 | 7.6 | 5.8 | 6.1 | 0.5 | 0.38 | G | |
| *Carex limosa* | 55 | 5.7 | 5.9 | 5.5 | 1.1 | 0.38 | G | |
| *Carex magellanica* | 6 | 11.2 | 5.9 | 5.2 | 0.4 | 0.46 | G | |
| *Carex pauciflora* | 30 | 15.8 | 10.7 | 4.8 | 1.1 | 0.46 | G | |
| *Carex rostrata* | 47 | 7.0 | 5.7 | 5.7 | 0.9 | 0.42 | G | |
| *Carex vaginata* | 3 | 48.0 | 22.3 | 5.9 | 0.1 | 0.42 | G | |
| *Chamaedaphne calyculata* | 13 | 14.3 | 6.7 | 3.9 | 0.3 | 0.58 | G | |
| *Comarum palustre* | 6 | 13.1 | 9.2 | 6.1 | 0.2 | 0.38 | G | |
| *Dactylorhiza maculata* | 1 | 15.0 | | 5.3 | | 0.35 | s | |
| *Dactylorhiza traunsteineri* | - | | | | | 0.19 | s | |
| *Deschampsia cespitosa* | 2 | 23.5 | 2.4 | 5.8 | 0.1 | 0.27 | s | M |
| *Deschampsia flexuosa* | 5* | | | 5.9 | 0.1 | 0.33 | s | F |
| *Drosera anglica* | 15 | 0.7 | 2.3 | 4.8 | 0.5 | 0.38 | G | |
| *Drosera rotundifolia* | 57 | 15.2 | 13.7 | 4.9 | 1.1 | 0.62 | G | |
| *Dryopteris carthusiana* | 13* | | | 6.0 | 0.3 | 0.27 | s | F |
| *Epilobium angustifolium* | 3 | 15.7 | 1.9 | 6.2 | 0.1 | 0.35 | s | M |
| *Epilobium palustre* | 14 | 9.3 | 5.6 | 6.0 | 0.7 | 0.31 | s | |
| *Empetrum nigrum* | 66 | 18.4 | 8.2 | 5.5 | 1.0 | 0.46 | G | F |

| | | | | | | | | |
|---|---|---|---|---|---|---|---|---|
| *Equisetum fluviatile* | 58 | 8.1 | 6.7 | 5.9 | 0.4 | 0.46 | G | A |
| *Equisetum sylvaticum* | 5 | 21.8 | 18.7 | 6.3 | 0.1 | 0.38 | G | F |
| *Eriophorum angustifolium* | 22 | 6.3 | 5.5 | 5.7 | 0.7 | 0.38 | G | |
| *Eriophorum latifolium* | 15 | 9.1 | 7.2 | 6.4 | 0.4 | 0.19 | s | |
| *Eriophorum vaginatum* | 33 | 12.1 | 6.5 | 4.3 | 0.5 | 0.54 | G | |
| *Filipendula ulmaria* | 8 | 15.1 | 7.2 | 6.2 | 0.2 | 0.31 | s | M |
| *Gymnocarpium dryopteris* | 17* | | | 5.8 | 0.4 | 0.27 | s | F |
| *Listera cordata* | 4* | | | 5.6 | 0.4 | 0.35 | s | |
| *Lysimachia europaea* | 14 | 17.8 | 11.8 | 5.5 | 0.9 | 0.35 | s | F |
| *Maianthemum bifolium* | 1 | 16.0 | | 6.2 | | 0.27 | s | F |
| *Melampyrum pratense* | 5 | 16.2 | 19.2 | 4.6 | 1.1 | 0.50 | G | F |
| *Menyanthes trifoliata* | 109 | 10.5 | 10.4 | 5.6 | 0.8 | 0.35 | s | |
| *Molinia caerulea* | 104 | 12.7 | 10.1 | 6.2 | 0.3 | 0.37 | G | |
| *Moneses uniflora* | 1* | | | 4.5 | | 0.35 | s | F |
| *Paris quadrifolia* | 8* | | | 6.2 | 0.3 | 0.27 | s | F |
| *Parnassia palustris* | 8 | 12.2 | 6.3 | 5.9 | 0.1 | 0.31 | s | |
| *Pedicularis sceptrum-carolinum* | - | | | | | 0.27 | s | |
| *Pyrola minor* | 1 | 13.0 | | 6.7 | | 0.27 | s | F |
| *Pyrola rotundifolia* | 2 | 15.9 | 0.8 | 6.2 | 0.1 | 0.40 | G | F |
| *Rhododendron tomentosum* | 14 | 14.3 | 8.3 | 4.2 | 0.7 | 0.46 | G | |
| *Rhynchosphora alba* | 6 | 1.3 | 4.9 | 4.5 | 0.7 | 0.38 | G | |
| *Rubus chamaemorus* | 32 | 13.8 | 6.3 | 4.4 | 1.0 | 0.50 | G | |
| *Salix myrsinifolia* | 3 | 5.9 | 5.5 | 6.5 | 0.1 | 0.42 | G | F |
| *Salix myrtilloides* | 4 | 22.4 | 24.5 | 5.8 | 0.4 | 0.54 | G | |
| *Salix phylicifolia* | 4 | 9.1 | 5.8 | 6.5 | 0.5 | 0.38 | G | F |
| *Scheuchzeria palustris* | 33 | 4.3 | 4.2 | 4.2 | 0.3 | 0.35 | s | |
| *Selaginella selaginoides* | 63 | 10.6 | 7.4 | 6.3 | 0.3 | 0.35 | s | |
| *Solidago virgaurea* | 14 | 15.5 | 5.8 | 6.1 | 0.4 | 0.42 | G | F |
| *Tofieldia pusilla* | 27 | 9.9 | 5.1 | 6.4 | 0.2 | 0.37 | G | |
| *Trichophorum alpinum* | 63 | 5.1 | 4.7 | 6.4 | 0.5 | 0.35 | s | |
| *Trichophorum cespitosum* | 65 | 8.7 | 5.9 | 6.0 | 0.7 | 0.40 | G | |
| *Utricularia minor* | 4 | 1.6 | 0.6 | 5.5 | 1.2 | 0.27 | s | |
| *Vaccinium microcarpum* | 40 | 15.4 | 8.9 | 5.3 | 1.1 | 0.46 | G | |
| *V. oxycoccos* | 114 | 15.5 | 12.0 | 5.4 | 0.9 | 0.54 | G | |
| *V. uliginosum* | 22 | 15.0 | 6.9 | 4.4 | 0.8 | 0.46 | G | |
| *V. vitis-idaea* | 10 | 17.2 | 14.4 | 6.3 | 0.2 | 0.46 | G | F |
| *Viola palustris* | 20* | | | 5.9 | 0.2 | 0.23 | s | |

## 2. Summary of results from SumF-tests

Summary of SumF-test results of differences in vegetation plot data between 1998 and 2018. We divided the vegetation plot data into four pH classes, based on pH in 1998. These four pH classes were treated separately. Results are given for all plant species and for bryophytes and vascular plants from separate test runs. The vegetation plot id was used as a blocked factor, and thus, testing focuses on pairwise differences in plot data between years. Monte Carlo permutation tests with 4999 runs was applied, and randomized runs' parameters are given with the probability (*p*) of observed SumF belonging to random distribution.

High pH = > 5.4

Intermediate pH = 4.6 – 5.4

Low pH = 4.3 – 4.6

Extremely low pH = < 4.3

| | SumF | Randomized SumF | | | |
|---|---|---|---|---|---|
| | Observed | Mean | Max | SD | p |
| **High pH** | | | | | |
| all species | 222.6 | 111.9 | 176.1 | 12.1 | 0.0002 |
| bryophytes | 68.4 | 48.5 | 77.1 | 5.9 | 0.0020 |
| vascular plants | 143.9 | 63.8 | 104.0 | 8.2 | 0.0002 |
| **Intermediate pH** | | | | | |
| all species | 133.5 | 79.5 | 126.5 | 9.8 | 0.0002 |
| bryophytes | 39.7 | 39.6 | 63.8 | 5.3 | 0.4704 |
| vascular plants | 81.5 | 40.8 | 71.7 | 7.1 | 0.0002 |
| **Low pH** | | | | | |
| all species | 105.1 | 59.3 | 100.2 | 8.5 | 0.0002 |
| bryophytes | 52.2 | 29.6 | 52.6 | 5.1 | 0.0006 |
| vascular plants | 35.6 | 28.6 | 53.0 | 5.2 | 0.0948 |
| **Extremely low pH** | | | | | |
| all species | 111.2 | 55.2 | 98.8 | 8.4 | 0.0002 |
| bryophytes | 64.5 | 30.5 | 56.1 | 5.4 | 0.0002 |
| vascular plants | 59.4 | 24.6 | 54.8 | 5.6 | 0.0002 |

## 3. Summary of results from Blocked Indicator Species Analysis (bISA)

Summary of results from Blocked Indicator Species Analysis (bISA; Dufrêne & Legendre 1997) assessing species differences between the 1998 and 2018 vegetation plot data. Four pH classes were treated separately. Only species with significant ($p < 0.05$) bISA values are shown. Vegetation plot id was used as a blocked factor. Monte Carlo permutation tests with 4999 runs were employed and parameters of randomized runs are given with the probability (p) of observed indicator value (IV) belonging to random distribution.

| Class | Year | Species | IV | IV Mean | IV SD | p |
|---|---|---|---|---|---|---|
| High pH | 1998 | *Trichophorum alpinum* | 30.9 | 16.9 | 2.97 | 0.0008 |
| | | *Bryum weigelii* | 10.9 | 5.6 | 1.79 | 0.0304 |
| | 2018 | *Drosera rotundifolia* | 48.6 | 21.7 | 3.8 | 0.0002 |
| | | *Menyanthes trifoliata* | 28.2 | 13.4 | 2.76 | 0.0002 |
| | | *Selaginella selaginoides* | 34.7 | 19 | 3.09 | 0.0004 |
| | | *Sphagnum warnstorfii* | 46.2 | 32.3 | 3.46 | 0.0022 |
| | | *Dicranum bonjeanii* | 24.2 | 13.4 | 2.71 | 0.0034 |
| | | *Trichophorum cespitosum* | 28.9 | 16.9 | 3.06 | 0.004 |
| | | *Angelica sylvestris* | 17 | 7 | 2.25 | 0.0048 |
| | | *Eriophorum vaginatum* | 30.4 | 21.1 | 3.29 | 0.0138 |
| | | *Molinia caerulea* | 41 | 31.1 | 3.67 | 0.0162 |
| | | *Empetrum nigrum* | 33.6 | 26 | 3.07 | 0.0228 |
| | | *Scheuchzeria palustris* | 12.3 | 6.1 | 2.12 | 0.0344 |
| Interm. pH | 1998 | *Trichophorum alpinum* | 42.3 | 21.2 | 3.42 | 0.0002 |
| | | *Sphagnum platyphyllum* | 11.3 | 5.7 | 1.83 | 0.0278 |
| | | *Drosera anglica* | 16.8 | 10.4 | 2.52 | 0.0362 |
| | 2018 | *Scheuchzeria palustris* | 45.1 | 22.6 | 3.38 | 0.0002 |
| | | *Trichophorum cespitosum* | 41.1 | 24.1 | 3.52 | 0.0004 |
| | | *Drosera rotundifolia* | 46.2 | 31.7 | 3.83 | 0.0044 |
| | | *Andromeda polifolia* | 51.1 | 42.9 | 3.07 | 0.0164 |
| | | *Sphagnum recurvum* agg. | 41.5 | 34.1 | 3.35 | 0.0338 |
| | | *Rhynchospora alba* | 19.7 | 13.1 | 2.85 | 0.0396 |
| | | *Straminergon stramineum* | 17.1 | 9.9 | 2.71 | 0.0414 |
| Low pH | 1998 | *Sphagnum annulatum* | 14.7 | 9.2 | 2.08 | 0.019 |
| | | *Trichophorum alpinum* | 13.3 | 5.9 | 2.41 | 0.0302 |
| | | *Menyanthes trifoliata* | 17.1 | 11.9 | 2.36 | 0.0352 |
| | 2018 | *Sphagnum* cf. *magellanicum* | 44.3 | 30.5 | 3.38 | 0.0008 |
| | | *Eriophorum vaginatum* | 46.9 | 30 | 3.96 | 0.0012 |
| | | *Andromeda polifolia* | 56.6 | 43.9 | 3.39 | 0.0018 |
| | | *Sphagnum recurvum* agg. | 49 | 37.6 | 3.74 | 0.0102 |
| | | *Carex pauciflora* | 28.4 | 18.7 | 3.41 | 0.0136 |
| | | *Sphagnum fuscum* | 24.6 | 17.5 | 2.84 | 0.0234 |

| | | | | | | |
|---|---|---|---|---|---|---|
| Extr. low pH | 1998 | *Carex limosa* | 27.7 | 17.4 | 2.88 | 0.0054 |
| | | *Sphagnum russowii* | 21.2 | 13.5 | 2.49 | 0.0104 |
| | 2018 | *Sphagnum recurvum* agg. | 60.1 | 42.1 | 3.43 | 0.0002 |
| | | *Drosera rotundifolia* | 49.5 | 28.8 | 3.73 | 0.0002 |
| | | *Sphagnum medium* coll. | 56 | 39.8 | 3.47 | 0.0008 |
| | | *Eriophorum vaginatum* | 54.5 | 37.9 | 3.88 | 0.001 |
| | | *Empetrum nigrum* | 25.5 | 18.4 | 2.15 | 0.0042 |
| | | *Carex pauciflora* | 33.4 | 23.6 | 3.28 | 0.0124 |

## References


Dufrêne, M., & Legendre, P. (1997). Species assemblages and indicator species: the need for a flexible asymmetrical approach. *Ecological monographs*, *67*(3), 345-366.

Eurola, S., Kaakinen, E., Saari, V., Huttunen, A., Kukko-oja, K., & Salonen, V. (2015). Sata suotyyppiä. Opas Suomen suokasvillisuuden tuntemiseen [A hundred mire site types. A guide to mire vegetation of Finland].
